# A cell cycle-controlled redox switch regulates the topoisomerase IV activity

**DOI:** 10.1101/gad.257030.114

**Published:** 2015-06-01

**Authors:** Sharath Narayanan, Balaganesh Janakiraman, Lokesh Kumar, Sunish Kumar Radhakrishnan

**Affiliations:** School of Biology, Indian Institute of Science Education and Research, Thiruvananthapuram 695016, Kerala, India

**Keywords:** cell cycle, chromosome segregation, topoisomerase IV, DNA decatenation, ClpXP proteolysis, intracellular redox, *Caulobacter crescentus*

## Abstract

Topoisomerase IV (topo IV), an essential factor during chromosome segregation, resolves the catenated chromosomes at the end of each replication cycle. How the decatenating activity of the topo IV is regulated during the early stages of the chromosome cycle despite being in continuous association with the chromosome remains poorly understood. Here we report a novel cell cycle-regulated protein in *Caulobacter crescentus*, NstA (negative switch for topo IV decatenation activity), that inhibits the decatenation activity of the topo IV during early stages of the cell cycle. We demonstrate that in *C. crescentus*, NstA acts by binding to the ParC DNA-binding subunit of topo IV. Most importantly, we uncover a dynamic oscillation of the intracellular redox state during the cell cycle, which correlates with and controls NstA activity. Thus, we propose that predetermined dynamic intracellular redox fluctuations may act as a global regulatory switch to control cellular development and cell cycle progression and may help retain pathogens in a suitable cell cycle state when encountering redox stress from the host immune response.

## Abstract (precis)

Narayanan et al. show in *C. crescentus* that NstA acts by binding to the ParC DNA-binding subunit of topoisomerase IV and inhibits its decatenation activity. They also uncover a dynamic oscillation of the intracellular redox state during the cell cycle, which correlates with and controls NstA activity.

All branches of life use topoisomerases to resolve the topological problems that arise during the process of chromosome replication (Champoux 2001). One of the major topological problems is the formation of catenated chromosomes, an intermolecular linkage between two replicated molecules of DNA (Sherratt 2003). In bacteria, chromosome catenation is particularly problematic due to the circular nature of the chromosomes. Such residual topological linkages of replicated sister chromatids, if not resolved, are lethal to dividing cells (Espeli and Marians 2004). In bacteria, these catenated linkages are removed by the decatenation activity of one of the type II topoisomerases, topoisomerase IV (topo IV) (Kato et al. 1990; Adams et al. 1992). The DNA decatenation activity of the ATPase-dependent topo IV is stimulated by specifically recognizing the “left-handed” or positive crossovers (Charvin et al. 2003; Stone et al. 2003; Corbett et al. 2005) and promotes unlinking and proper segregation of the entangled sister chromatids into dividing daughter cells (Zechiedrich et al. 1997; Deibler et al. 2001; Espeli and Marians 2004; Wang and Shapiro 2004; Wang et al. 2013). The active topo IV complex is a heterotetramer of two subunits, ParC and ParE (Peng and Marians 1993b). While the ParE subunit contains the ATPase domain fueling the reaction, the ParC subunit is responsible for binding, cleavage, and religation of dsDNA (Peng and Marians 1993b). Genes encoding both the ParC and ParE subunits of topo IV are essential in *Escherichia coli*, *Salmonella typhimurium*, and *Caulobacter crescentus* (Adams et al. 1992; Peng and Marians 1993a; Zechiedrich and Cozzarelli 1995; Ward and Newton 1997). Moreover, topo IV and the other type II topoisomerase, DNA gyrase, are targets of the most commonly used quinolone antibiotics (Aldred et al. 2014).

The synchronizable bacterium *C. crescentus*, due to its clear separation of the S, G1, and G2–M phases, is a useful model for studying the processes of chromosome replication and segregation during the cell cycle. During the cell cycle, *C. crescentus* divides asymmetrically to give developmentally different daughter cells: a swarmer cell that is in a G1 phase, in which no division or chromosome replication happens, and a stalked cell that is in S phase, engaged in chromosome replication and cell division (Curtis and Brun 2010). The swarmer cell has to terminally differentiate into a stalked cell (G1 → S transition) in order to enter the replication–division cycle. Several regulatory proteins—most importantly, the activity of the DNA-binding response regulator CtrA (Quon et al. 1998) and the protease ClpXP (Domian et al. 1997; Jenal and Fuchs 1998; Chien et al. 2007)—govern the precise execution of the cell cycle. In the G1 cells, the active and phosphosphorylated form of CtrA, CtrA∼P, inhibits initiation of chromosome replication by binding to the origin of replication (*ori*), while, during the G1 → S transition, CtrA proteolysis by ClpXP is activated, liberating the *ori* and thus facilitating initiation of chromosome replication (Quon et al. 1998). In addition to regulating the levels of CtrA, the ClpXP protease also controls the abundance of a plethora of developmentally important regulatory proteins during the cell cycle, including the toxin SocB, which regulates the chromosome replication elongation under stress conditions in the absence of its antitoxin, SocA, by binding to the β-sliding clamp (Aakre et al. 2013). Interestingly, the removal of ClpXP is no longer lethal in the absence of SocB. Nevertheless, the *socB*^−^ *clpXP*^−^ mutant is reported to be filamentous (Aakre et al. 2013). Therefore, it is conceivable that there are other developmentally important ClpXP targets that are not degraded in the absence of ClpXP and give rise to the developmental defect in the *socB*^−^ *clpXP*^−^ mutant.

In *C. crescentus*, upon initiation of the chromosome replication during the G1 → S transition, the polar *ori* is replicated, segregated, and tethered to the opposite pole (Fig. 1A). The translocation and subsequent tethering of the *ori* to the opposite pole are brought about by the concerted activities of the partitioning proteins ParA and ParB, which bind to the *cis*-acting origin-proximal *parS* site on the chromosome and the PopZ polar anchoring complex (Mohl et al. 2001; Figge et al. 2003; Thanbichler and Shapiro 2008; Toro et al. 2008; Shapiro et al. 2009; Shebelut et al. 2010; Lim et al. 2014). As in most bacteria, in *C. crescentus*, the ParC subunit of topo IV loads onto the moving replisome coincident with the initiation of replication and moves along with the replisome toward the termination sites (Fig. 1A; Wang and Shapiro 2004). Unlike ParC, the ParE subunit is dispersed (Fig. 1A; Wang and Shapiro 2004). Nevertheless, the localization of ParC is dependent on the presence of active ParE (Wang and Shapiro 2004). The activity and/or localization of topo IV is thought to be dependent on the divisome component FtsK (Espeli et al. 2003a). In *E. coli*, a physical interaction between FtsK and topo IV has been shown to stimulate the in vitro activity of topo IV (Espeli et al. 2003a; Bigot and Marians 2010). While, in *C. crescentus*, the localization of the ParC subunit to the replisome is found to be mediated through FtsK, no physical interaction has been demonstrated (Wang et al. 2006). The formation of a functional topo IV complex very early during the chromosome cycle, together with the knowledge that the decatenation activity of topo IV predominates during the late stages of chromosome cycle (Espeli et al. 2003b; Wang and Shapiro 2004), raises the questions of whether mechanisms exist that restrict the decatenation activity of topo IV complex to the end of the *C. crescentus* cell cycle and how this is accomplished.

**Figure 1.**
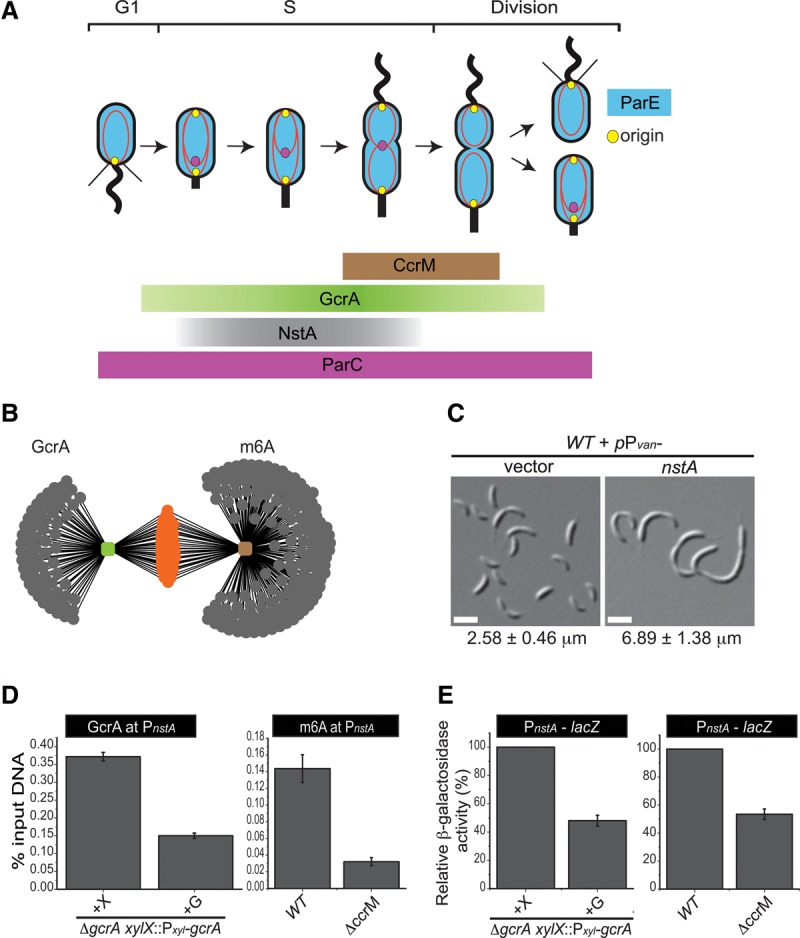
S-phase-specific developmental regulators in *C. crescentus*. (*A*) Schematic showing the cell cycle abundance of the S-phase-specific regulatory molecules CcrM (brown) and GcrA (green) and the topo IV regulator NstA (negative switch for topo IV decatenation activity) (gray). Also shown are the replication and sequestration of the newly replicated origin (yellow) and the presence of ParC (magenta) and ParE (blue) during the cell cycle in *C. crescentus*. (*B*) Network image showing putative promoters (gray dots) regulated by CcrM, detected using m6A (brown) and GcrA (green), as inferred from ChIP-seq (chromatin immunoprecipitation [ChIP] combined with “deep” sequencing) results of wild-type *C. crescentus* (Fioravanti et al. 2013). The common targets between m6A and GcrA are shown in orange. (*C*) Differential interference contrast (DIC) microscopy images of wild-type (WT) *C. crescentus* cells harboring the high copy vector (pMT335) or overexpressing *nstA* from the vanillate-inducible promoter P_*van*_ on pMT335. Vanillate (0.5 mM) induction was done for 6 h. Mean cell size ± SD of at least 200 cells is given at the *bottom* of the image. Bar, 2 μm. (*D*) Data of qChIP (quantitative ChIP) experiments showing the occupancy of GcrA and m6A at P_*nstA*_ in *gcrA*-depleted or *ccrM*-deleted cells. (*E*) Relative percentage of β-galactosidase activity of the P*_nstA_-lacZ* reporter in wild-type, *gcrA*-depleted, and *ccrM*-deleted cells. The *gcrA xylX*::P_*xyl*_-*gcrA* cells were grown in the presence of xylose (+X) or glucose (+G) for 9 h. The values, ±SE, denoted in *D* and *E* are the average of three independent experiments.

Here, we identify NstA (negative switch for topo IV decatenation activity), a cell cycle-dependent regulator, and report that it functions by directly inhibiting the decatenation activity of topo IV during early stages of the cell cycle in *C. crescentus*. We demonstrate that NstA binds the ParC subunit of the topo IV complex. Moreover, we show that NstA is confined to the early stages of the cell cycle by a combination of S-phase-specific synthesis and ClpXP-mediated degradation. We further provide evidence that the activity of NstA peaks with the intracellular oxidation of the cytoplasm. Above all, our experiments for the first time show that the intracellular oxidation–reduction (redox) state is regulated during the bacterial cell cycle. These findings further our understanding of the complex interplay between the redox state and the cell cycle, with major implications for the cell cycle control of bacterial pathogens exposed to the chemical warfare of the host immune system.

## Results

### A novel genetic approach to identify S-phase-specific developmental regulators

The cell cycle-regulated proteins GcrA and CcrM are two important transcriptional regulators that are primarily known to target promoters that fire in S phase of the *C. crescentus* cell cycle (Mohapatra et al. 2014). The cell cycle-regulated methyltransferase CcrM facilitates the binding of the global transcriptional regulator GcrA to its preferred target promoters (Fioravanti et al. 2013) via N6 adenine methylation (m6A) of nearly all of the 4000 5′-GANTC-3′ sites in the *C. crescentus* genome. Following replication, these sites are hemimethylated, and CcrM remethylates them once it accumulates in late S phase (Fig. 1A; Zweiger et al. 1994; Stephens et al. 1996). GcrA, synthesized in early S phase, preferentially binds and activates target promoters carrying such m6A marks. Mutation of the methylation motif in these promoters or inactivation of CcrM impairs binding of GcrA to these promoters (Fioravanti et al. 2013). Several important cell cycle proteins are directly regulated by GcrA/CcrM, such as the master cell cycle regulator CtrA, the cytokinetic tubulin FtsZ, the cell division positioning factor MipZ, the FtsN division protein, and the PodJ polarity determinant (Laub et al. 2000; Viollier et al. 2002; Thanbichler and Shapiro 2006; Fioravanti et al. 2013; Gonzalez and Collier 2013). Reasoning that other cell cycle factors operating specifically during the S phase are also under GcrA/CcrM control, we analyzed several uncharacterized GcrA/CcrM targets identified in ChIP-seq (chromatin immunoprecipitation [ChIP] combined with “deep” sequencing) experiments using antibodies to GcrA and m6A (Fioravanti et al. 2013). Bioinformatic analysis using a low-stringency cutoff identified 97 putative promoters as targets of GcrA/CcrM (Fig. 1B). Of these, 68 targets had upstream “GANTC” sites (Supplemental Table S1; Fioravanti et al. 2013).

Next, we systematically surveyed the uncharacterized GcrA/CcrM target genes for growth defects upon their overexpression from the vanillate-inducible promoter (P_*van*_) on a high copy vector throughout the wild-type *C. crescentus* cell cycle. We focused on CCNA_03091, which yielded a severe developmental defect upon overexpression. CCNA_03091, referred to here as *nstA*, is predicted to encode an uncharacterized protein of 66 residues. Overexpression of *nstA* for 6 h produced filamentous cells compared with the control cells harboring the vector alone (Fig. 1C; Supplemental Table S2). Thus, constitutive overexpression of NstA disturbs the cell division cycle.

### Temporal regulation of NstA abundance

To confirm that NstA expression is induced in S phase, we used quantitative ChIP (qChIP) experiments to show that GcrA and CcrM indeed bind to the *nstA* promoter (P_*nstA*_) in vivo. Five hours of depletion of GcrA using a strain harboring the xylose-inducible P*_xyl_-gcrA* as the only copy of *gcrA* on the chromosome (Holtzendorff et al. 2004) resulted in ∼60% reduction in the occupancy GcrA on P_*nstA*_ (Fig. 1D). Moreover, antibodies to m6A precipitated P_*nstA*_ far less efficiently (∼85% reduction) from chromatin of *ccrM*-deleted cells versus wild-type cells (Fig. 1D). Consistent with these results, β-galactosidase production from *lacZ*-based reporter fusions in which the promoter of *nstA* was fused to the promoterless *lacZ* reporter gene (P_*nstA*_-*lacZ*) on a low copy plasmid was reduced in the absence of GcrA and CcrM, showing that they positively influenced the transcription from P_*nstA*_. Five-hour depletion of GcrA decreased the P_*nstA*_-*lacZ* activity by ∼35% (Supplemental Fig. S1), while a depletion for 9 h decreased the activity by ∼50% (Fig. 1E). Deletion of *ccrM* decreased the activity of P_*nstA*_-*lacZ* by ∼50% (Fig. 1E) compared with the wild type. The persistence of previously synthesized GcrA and residual *gcrA* synthesis from P_*xyl*_ even in the repressed state along with high affinity of GcrA for P_*nstA*_ likely explains the residual promoter activity.

**Figure 2.**
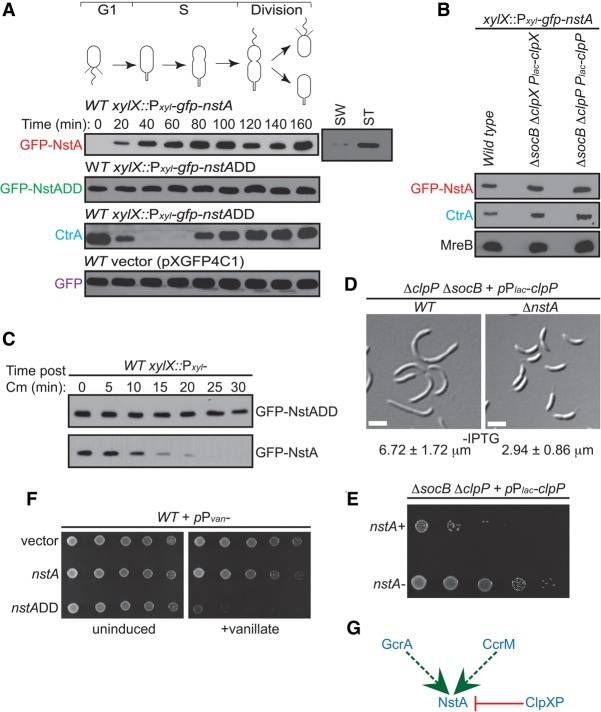
Regulation of NstA abundance during the cell cycle by the ClpXP protease. (*A*) Immunoblot analyses in synchronized populations of *C. crescentus* to determine the relative abundance of GFP-NstA, GFP-NstADD, CtrA, and GFP during the cell cycle. Wild-type (WT) cells harboring the plasmids for *gfp*-*nstA*, *gfp*-*nstADD*, and *gfp* at the *xylX* locus on the chromosome were used. Cells were treated with 0.3% xylose. Levels of GFP-NstA in swarmer (SW) and stalked (ST) cell populations of resynchronized cells at 140 min are also shown. (*B*) Immunoblots of GFP-NstA, CtrA, and MreB in wild-type cells and in Δ*socB* cells depleted for *clpX* or *clpP*. The strains were not treated with IPTG, the inducer for the P_*lac*_ promoter. MreB was used as the loading control. (*C*) Immunoblots showing the difference in stability of GFP-NstA and GFP-NstADD. Expression of *gfp-nstA* or *gfp-nstADD* was induced with 0.3% xylose for 4 h prior to the inhibition of translation by chloramphenicol (Cm) treatment. The abundance of GFP-NstA or GFP-NstADD was monitored over time as indicated. (*D*) DIC microscopy images of Δ*socB* cells depleted for *clpP* in the presence and absence of *nstA.* Cells were not treated with IPTG. Mean cell size ± SD of at least 200 cells is given at the *bottom* of the image. Bar, 2 μm. (*E*) Dilution plate showing the growth of cells from *D*. Fivefold serial dilutions of cells from *D* were spotted onto medium without IPTG. (*F*) Growth of wild-type strains harboring the high copy vector (pMT335) and expressing *nstA* or *nstADD* from the P_*van*_ promoter on pMT335. Fivefold dilutions of the indicated strains were spotted onto medium with or without the inducer vanillate (0.5 mM). (*G*) Schematic summarizing the transcriptional and post-translational regulation of NstA. (Dashed green line) Positive transcriptional regulation by GcrA and CcrM; (red line) negative post-translational regulation by the protease ClpXP.

To analyze whether NstA abundance during the cell cycle might be regulated at the level of protein stability, we used wild-type *C. crescentus* cells expressing a nonfunctional N-terminal GFP fusion to NstA whose gene was integrated at the chromosomal *xylX* locus and thereby was put under the control of a xylose-inducible promoter (*xylX*::P*_xyl_-gfp-nstA*). Immunoblotting revealed that GFP-NstA could not be detected in the G1 cells but accumulated upon the G1 → S transition, and, interestingly, the protein levels dropped in the late S phase or dividing cells (Fig. 2A; Supplemental Fig. S2). The abundance of GFP alone was invariant throughout the cell cycle in wild-type cells, ruling out the influence of GFP on the cell cycle abundance of GFP-NstA (Fig. 2A). NstA features two nonpolar amino acid residues (Ala–Ala) at the penultimate C-terminal position, a typical signature of ClpXP protease substrates (Domian et al. 1997; Gottesman et al. 1998; Chien et al. 2007). To test whether the two terminal nonpolar residues are important determinants of NstA abundance, Ala–Ala were mutated into aspartate residues Asp–Asp (NstADD). A construct encoding such engineered NstA fused to GFP at the N terminus was integrated at the *xylX* locus (*xylX::*P*_xyl_-gfp-nstA*DD). Immunoblotting revealed that the GFP-NstADD protein, like untagged GFP but unlike wild-type GFP-NstA, was stable throughout the cell cycle, as expected for ClpXP substrates that have been stabilized (Fig. 2A; Domian et al. 1997; Radhakrishnan et al. 2010). The degradation of other well-defined ClpXP substrates, such as CtrA (Domian et al. 1997), was not affected in the cells producing NstADD (Fig. 2A), indicating that, under these conditions, NstADD does not sequester ClpXP. Moreover, the abundance of GFP-NstADD in unsynchronized cells was higher than that of GFP-NstA (Supplemental Fig. S3), and GFP-NstADD exhibited higher stability than GFP-NstA in antibiotic chase experiments (Fig. 2C). Above all, the abundance of GFP-NstA was elevated, and its stability increased in cells in which either ClpX or ClpP was depleted (Fig. 2B; Supplemental Fig. S4A,B; Aakre et al. 2013).

In sum, *C. crescentus* cells rely on transcriptional regulation of *nstA* through GcrA and CcrM and a post-translational regulation of NstA abundance through ClpXP to maintain a tight control on the abundance of NstA during S phase of the cell cycle (Figs. 1A, 2G).

### Regulation of nstA production and abundance is indispensable for proper cell cycle progression

Next, we analyzed whether the degradation of NstA by ClpXP is important for the normal cell cycle progression. To this end, we compared the phenotype of *clpP socB* double-mutant cells in the presence or absence of NstA. Deletion of the gene encoding the β-sliding clamp inhibitor SocB only partially alleviates the developmental defects and viability of the cells depleted for *clpP.* Surprisingly, deletion of *nstA* and *socB* together in the cells depleted of *clpP* improved colony formation and completely alleviated the developmental defects of cell filamentation and motility (Fig. 2D,E; Supplemental Fig. S4C). Thus, NstA has negative effects when ClpP is absent. Moreover, expression of low levels of NstADD from the *xylX* locus (*xylX::P_xyl_-nstADD*) yielded filamentous cells (Supplemental Fig. S5). Furthermore, overproduction of NstADD induced toxicity in wild-type cells (Fig. 2F). Together, these results demonstrated that NstA is a substrate of ClpXP and that the timely control of *nstA* production and abundance was important for the proper cell cycle progression in *C. crescentus*.

### NstA operates by binding to the ParC subunit of topo IV

**Figure 3.**
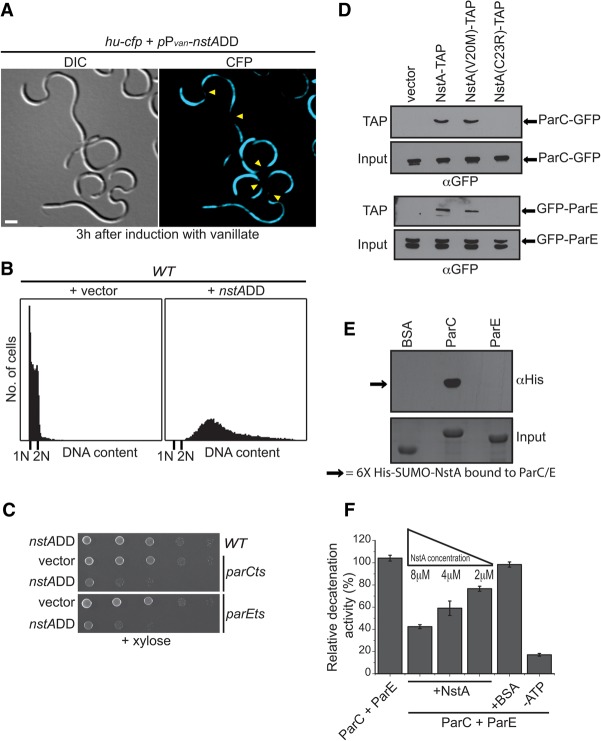
NstA inhibits the activity of topo IV. (*A*) DIC and fluorescence microscopy images of wild-type cells overexpressing *nstADD* from the P_*van*_ promoter on the high copy vector pMT335 and harboring the nonspecific chromosome-binding protein HU fused at the C terminus with the cyan fluorescent protein (CFP) expressed from the native chromosomal locus (*hu*::*hu-cfp*). The cells were induced with 0.5 mM vanillate for 3 h to induce the production of NstADD. Yellow arrowheads denote the chromosome-free regions. Bar, 2 μm. (*B*) Flow cytometry profiles to show the DNA content of wild-type (WT) cells overexpressing *nstADD* in comparison with cells harboring the empty vector alone. Overexpression of *nstADD* was done as described in *A*. (*C*) Growth of the wild-type cells and the temperature-sensitive *parC*ts and *parE*ts mutants in the presence and absence of *nstADD*. The expression of nstADD was from the chromosomal *xylX* locus (*xylX::*P_*xyl*_-*nstADD*). Fivefold dilutions of the indicated strains were spotted onto medium containing 0.3% xylose. The cells were grown at permissive temperature. (*D*) Immunoblots of TAP samples of extracts from wild-type cells harboring *parC-gfp* (*top* panel) or *gfp-parE* (*bottom* panel) expressing NstA-TAP, NstA(V20M)-TAP, or NstA(C23R)-TAP from P_*van*_ on pMT335. The extracts of *parC-gfp* or *gfp-parE* strains with the empty vector were used as a control. Anti-GFP (αGFP) was used for detection. (*E*) Far-Western analysis using purified ParC, ParE, and His_6_-SUMO-NstA. (*Top* panel) A blot containing 0.1 nM ParC, ParE, or BSA was incubated with 25 nM purified His_6_-SUMO-NstA and further probed with monoclonal hexa-Histidine antibody (αHis). (*Bottom* panel) A Coomassie brilliant blue-stained gel with 0.1 nM BSA, ParC, and ParE is shown as the input control. (*F*) Relative in vitro DNA decatenation activity of topo IV (ParC + ParE) in the presence of various concentrations of NstA. The reactions were carried out with either ParC and ParE alone or together with increasing concentrations of NstA as indicated. Reactions with BSA equivalent to 8 μM NstA or without ATP were used as controls. The data are the average of three independent experiments ±SE.

The filamentous phenotype of the cells overproducing NstADD suggested a defect in the cell division cycle. To investigate the molecular mechanism by which overproduction of NstADD induced toxicity in wild-type *C. crescentus*, we stained chromosomes in NstADD-overproducing cells with DAPI after fixation and observed DNA-free regions, pointing to a defect in segregation (Supplemental Fig. S6). Live-cell imaging with *hu-cfp* cells also revealed the presence of several chromosome-free regions in the NstADD-overproducing cells (Fig. 3A). In contrast, such nucleoid-free regions were not found in the filamentous cells depleted for the divisome component FtsZ (Supplemental Fig. S6; Wang et al. 2001). Moreover, the nucleoid-free regions in the NstADD-overproducing cells were strikingly similar to those reported for the cells harboring mutations in the ParC or ParE subunit of topo IV (Ward and Newton 1997). These data indicated that NstA might be antagonizing the activity of topo IV though ParC and/or ParE. Thus, we hypothesized that even basal levels of NstADD might be lethal to the cells having crippled topo IV activity, such as those harboring a partial loss-of-function mutation in the genes encoding ParC or ParE. To test this, we expressed *xylX::P_xyl_-nstADD* in cells harboring temperature-sensitive mutations in either *parC* (*parC*ts) or *parE* (*parE*ts) (Ward and Newton 1997). As expected, production of NstADD from *xylX::P_xyl_-nstADD* substantially decreased the viability of both *parC*ts and *parE*ts mutants even at permissive temperature compared with wild-type cells (Fig. 3C). Furthermore, to check whether NstA interacts with the topo IV subunits in vivo, copurification experiments using C-terminal tandem affinity purification (TAP)-tagged NstA were done and established that NstA pulled down the ParC and ParE subunits of the topo IV complex (Fig. 3D). Additionally, in vitro Far-Western experiments using purified NstA, ParC, and ParE showed that NstA and the ParC subunit of topo IV interact directly (Fig. 3E). To analyze whether NstA negatively influenced the decatenation activity of topo IV, we reconstituted DNA decatenation assays in vitro using purified NstA, ParC, and ParE and found that the DNA decatenation potential of topo IV decreased with increasing concentrations of the purified NstA when compared with the reaction lacking NstA (Fig. 3F; Supplemental Fig. S7). These results established that NstA acts as a negative regulator of the DNA decatenation activity of topo IV during S phase by specifically binding to the ParC subunit of topo IV.

### Intermolecular cysteine disulfide controls the activity of NstA

**Figure 4.**
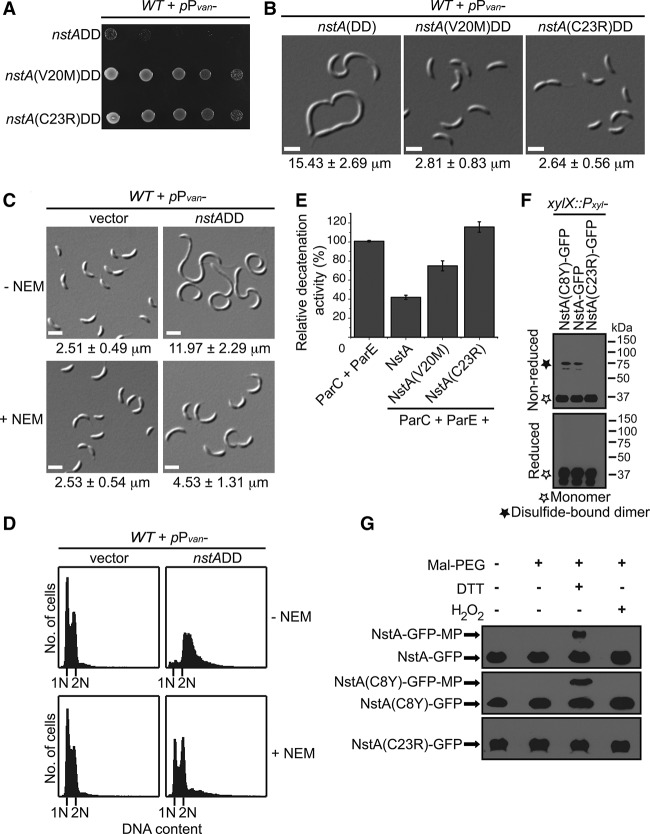
Cysteine disulfide-dependent activity of NstA. (*A*) Growth of wild-type (WT) cells overexpressing *nstADD*, *nstA*(V20M)DD, and *nstA*(C23R)DD from P_*van*_ on pMT335. Fivefold diluted cultures were spotted onto medium containing 0.5 mM vanillate. (*B*) DIC microscopy images of cells in *A*. (*C*) DIC microscopy images of wild-type cells overexpressing *nstA*DD from P_*van*_ on pMT335 in the presence or absence of N-ethylmaleimide (NEM). Cells were pretreated with 7.5 μM NEM for 2 h prior to the addition of 0.25 mM vanillate. (*D*) Flow cytometry profiles to show the DNA content of cells in *C*. (*E*) Relative in vitro DNA decatenation activity of topo IV (ParC + ParE) in the presence of purified NstA, NstA(V20M), and NstA(C23R). (*F*) Immunoblots of reducing and nonreducing SDS-PAGE of NstA-GFP, NstA(C8Y)-GFP, and NstA(C23R)-GFP expressed from the *xylX* locus on the chromosome. (*G*) Immunoblots of NEM and maleimide-PEG (MP) modifications. The samples were extracted in the presence of excess NEM that immediately blocks all free thiol groups. Subsequently, disulfide bridges were reduced by the addition of DTT, and the newly formed thiol groups from the reduction of the disulfide bonds by DTT were modified with MP (molecular weight 5 kDa). Increased-molecular-weight form [NstA-GFP-MP or NstA(C8Y)-GFP-MP] denotes the existence of disulfide bridges. No disulfide bridge was formed in NstA(C23R), leading to the absence of higher-molecular-weight forms. Mean cell size ± SD of at least 200 cells is given at the *bottom* of the images in *B* and *C*.

To identify residues that are important for the activity of NstA, we mutagenized the NstADD-coding sequence and sought suppressor mutations that have lost the lethal overexpression phenotype. Intragenic suppressor mutations in *nstA*DD, Q2R, T5I, F7L, C8Y, D12G, V20M, C23R, L36F, E47K, and F56L were obtained (Fig. 4A; Supplemental Fig. S8A). Overproduction of the Q2R, T5I, C8Y, D12G, L36F, E47K, and F56L still induced mild or severe cell elongation in wild-type *C. crescentus* but did not affect the viability (Supplemental Fig. S8A,D), implying that these mutants might have a partial loss of function in activity. Interestingly two of the mutant proteins, NstA(V20M), and NstA(C23R), displayed a complete loss-of-function phenotype upon overproduction in wild-type cells (Fig. 4A,B; Supplemental Fig. S8B). Immunoblot experiments confirmed that the loss of function was not due to a decrease in NstA levels (Supplemental Fig. S8C).

In vivo copurification experiments showed that NstA(C23R) could no longer interact with the topo IV complex, while the NstA(V20M) mutant was bound to the ParC/ParE subunits, akin to wild-type NstA (Fig. 3D). Notwithstanding, from the in vitro DNA decatenation experiments, it was evident that NstA(V20M) had a reduced inhibitory effect on the decatenation activity of topo IV compared with the wild-type version (Fig. 4E). Nevertheless, the NstA(C23R) mutant did not show any inhibitory activity on topo IV in vitro (Fig. 4E). These experiments hinted at the importance of the cysteine residues for the activity of NstA.

To explore whether the activity of NstA is triggered through cysteine disulfide bond formation, we treated the cells overexpressing NstADD with N-ethylmaleimide (NEM). NEM is a thiol-reactive agent preventing cysteine disulfide bond formation in proteins or peptides by alkylating the sulfhydryls of the cysteine residues (Smyth et al. 1964; Gorin et al. 1966). Interestingly, the cells overexpressing NstADD that were treated with NEM showed a reduced cell elongation phenotype when compared with NstADD-overproducing cells that were not treated with NEM (Fig. 4C). The chromosome-free regions seen in the cells overexpressing NstADD were reduced in the presence of NEM and were mildly accentuated in the cells overexpressing nonstabilized NstA in the presence of the oxidizing agent hydrogen peroxide (Supplemental Fig. S9A,B). More importantly, the accumulation of chromosomes in the cells overproducing NstADD was strongly reduced in the presence of NEM (Fig. 4D). To avoid the possibility that the reversal is due to a general effect of NEM on the chromosome replication cycle, a concentration of NEM (7.5 μM) that did not significantly affect the chromosome replication in synchronized populations of wild-type cells carrying the vector or a mutant form of NstA, NstA(C23R)DD (Supplemental Fig. S10A,B), was used. Moreover, the effect of 7.5 μM NEM on the growth of wild-type *C. crescentus* was minimal (Supplemental Fig. S11A). Immunoblot experiments demonstrated that 7.5 μM NEM did not affect the stability of NstA (Supplemental Fig. S11C). Nevertheless, addition of 7.5 μM NEM decreased the amount of dimeric NstA-seGFP in cells overproducing NstA-seGFP (Supplemental Fig. S11B). The decrease in filamentation was not seen in cells overexpressing the cell division inhibitor KidODD (Supplemental Fig. S11D; Radhakrishnan et al. 2010), ruling out the possibility that the decrease in filamentation of the NstADD-overexpressing cells was due to a general slowdown of growth in the presence of NEM. Furthermore, the inhibitory activity of NstA in vitro against topo IV decreased in the presence of DTT (Supplemental Fig. S12). These experiments suggested that the cysteine disulfide bond formation might be important for the activity of NstA.

As we obtained the NstA toxicity suppressor mutations in two cysteine residues of NstA, C8 and C23, we went on to investigate whether both or either of the cysteines is involved in an intermolecular or intramolecular cysteine disulfide bond formation that activates NstA. To test this, we analyzed functional C-terminal GFP versions of wild-type NstA, NstA(C8Y), and NstA(C23R) proteins expressed in wild-type *C. crescentus* under nonreducing conditions. Our experiments showed that wild-type NstA and the mutant NstA(C8Y) could form higher-molecular-weight bands, corresponding to a dimeric form of NstA, under nonreducing conditions (Fig. 4F). This indicated that the C23 residue in NstA might alone be involved in the formation of an active NstA dimer through intermolecular disulfide bridges. To further validate this observation, we analyzed the sulfhydryl availability in NstA for alkylation by NEM and maleimide-PEG (MP). Here, MP specifically binds to the thiols generated as a result of DTT treatment, leading to the formation of increased-molecular-weight forms and providing evidence for the existence of disulfide bridges in proteins. While increased-molecular-weight forms (NstA-GFP-MP) were found in the cell lysates of NstA-GFP-expressing or NstA(C8Y)-GFP-expressing strains treated with DTT and MP, they were not present in the lysates of NstA(C23R)-GFP-expressing cells treated alike (Fig. 4G). Moreover, the increased-molecular-weight forms were not present in untreated samples or when the disulfides were stabilized with H_2_O_2_ (Fig. 4G). These experiments suggested that it is the dimeric form of NstA—formed by a C23-dependent intermolecular disulfide bond—that is active.

### A dynamic intracellular redox during cell cycle

**Figure 5.**
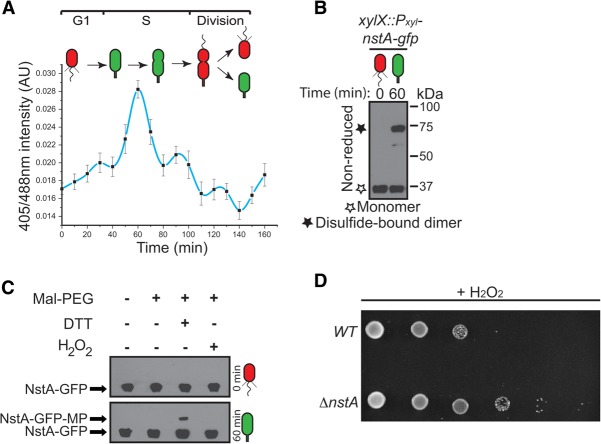
Redox-dependent regulation of the cell cycle. (*A*) Ratios (405/488 nm) of roGFP2 during the cell cycle in a synchronized population of wild-type *C. crescentus* expressing *rogfp2* from the chromosomal *xylX* locus (*xylX*::P_*xyl*_-*rogfp2*). The cells were treated with 0.3% xylose to induce the production of roGFP2. The data are an average of eight independent experiments ±SE. (*B*) Immunoblots of nonreducing SDS-PAGE of NstA-GFP in synchronized populations of cells at 0 and 60 min after synchronization. *nstA-gfp* was expressed from the *xylX* locus (*xylX*::P_*xyl*_-*nstA-gfp*). (*C*) Immunoblots of NEM and MP modifications of synchronized samples of the *xylX::*P*_xyl_-nstA-gfp* collected at 0 and 60 min after synchronization. The samples were treated as described in Figure 4G. The increased-molecular-weight form (NstA-GFP-MP) in the cells at 60 min denotes the existence of disulfide bridges while the cytoplasm is in an oxidized state during cell cycle. No disulfide bridge was formed in the reduced cytoplasm at 0 min, leading to the absence of higher-molecular-weight forms of NstA-GFP. (*D*) Growth of wild-type (WT) and Δ*nstA* cells in the presence of the oxidizing agent H_2_O_2_. Sixfold diluted cultures were spotted onto medium containing 40 µM H_2_O_2_. (*A*–*C*) (Red) Reduced cytoplasm; (green) oxidized cytoplasm. Bar, 2 μm.

It is well established that redox environment influences cysteine disulfide bond formations (Chakravarthi et al. 2006). Thus, we hypothesized that a cell cycle-controlled switch in the intracellular redox state might control NstA activity by disulfide bond formation. To test this, we explored the possibility that the intracellular redox state fluctuates during the *C. crescentus* cell cycle. To this end, we integrated a gene encoding a redox-sensitive derivative of GFP, *rogfp2*, at the *xylX* locus on the chromosome (*xylX::*P_*xyl*_-*rogfp2*) of wild-type *C. crescentus*. The redox-sensing roGFP2 with its engineered cysteines, S147C and Q204C, can be used to monitor intracellular redox states (Hanson et al. 2004; Bhaskar et al. 2014). In an oxidized state, formation of a disulfide bond between these two cysteines shifts the excitation maximum of roGFP2 to 405 nm, and, concomitantly in a reduced state, due to the absence of the cysteine disulfide bonds, the roGFP2 gets excited maximally at 488 nm. Thus, the ratio of fluorescence intensities at 405 nm and 488 nm will report the redox state of the cell (Hanson et al. 2004; Bhaskar et al. 2014). The effectiveness of the expressed roGFP2 was tested in the reduced or oxidized conditions by using DTT or H_2_O_2_, respectively (Supplemental Fig. S13). We measured the intracellular redox state of wild-type *C. crescentus* expressing roGFP2 constitutively during the cell cycle in a synchronized population. Interestingly, the ratio of roGFP2 fluorescence revealed that the intracellular redox state fluctuates during the *C. crescentus* cell cycle (Fig. 5A). The G1 cells had a reduced cytoplasm. The change from the reduced to oxidized cytoplasmic state occurred concomitant with the G1 → S transition. The oxidized state was increasingly prevalent until the mid-S phase and then gradually decreased toward the late stages of S phase (Fig. 5A). The oxidized intracellular state coincided with the window in the cell cycle during which NstA was present and active. Moreover, the redox state influenced the cysteine-dependent dimerization of NstA-GFP (Fig. 5B,C; Supplemental Fig. S14). Finally, there was no inhibition of growth on Δ*nstA* cells under oxidizing conditions when compared with the wild-type, indicating that, in highly oxidized conditions, NstA may influence growth by down-regulating chromosome segregation through the inhibition of topo IV (Fig. 5D). These experiments bolstered the notion that NstA acts as a redox sensor whose activity is dependent on the oxidized state of the cytoplasm, thereby coupling the chromosome cycle to the intracellular redox state. Thus, our data for the first time demonstrate cyclic changes in the intracelluar redox state during a bacterial cell cycle.

## Discussion

The process of DNA replication invariably leads to the formation of catenated DNA due to the topological constraints that arise during the replication process (Sherratt 2003). In bacteria, the catenated circular chromosomes are resolved and properly segregated by the activity of topo IV (Kato et al. 1990; Adams et al. 1992). Previous research has shown that, although topo IV is loaded onto the chromosome coincident with the formation of the replisome, the DNA decatenation activity is predominant only during the late stages of the chromosome cycle (Sherratt 2003; Espeli and Marians 2004; Wang and Shapiro 2004). The question that we addressed in this study is how the decatenation activity of topo IV is prevented during the early stages of the chromosome cycle. Here, we demonstrated that, in *C. crescentus*, the cell cycle-regulated protein NstA is involved in down-regulating the decatenating activity of topo IV during the early stages the chromosome cycle. NstA achieves this function by directly binding to the ParC subunit of the topo IV complex.

**Figure 6.**
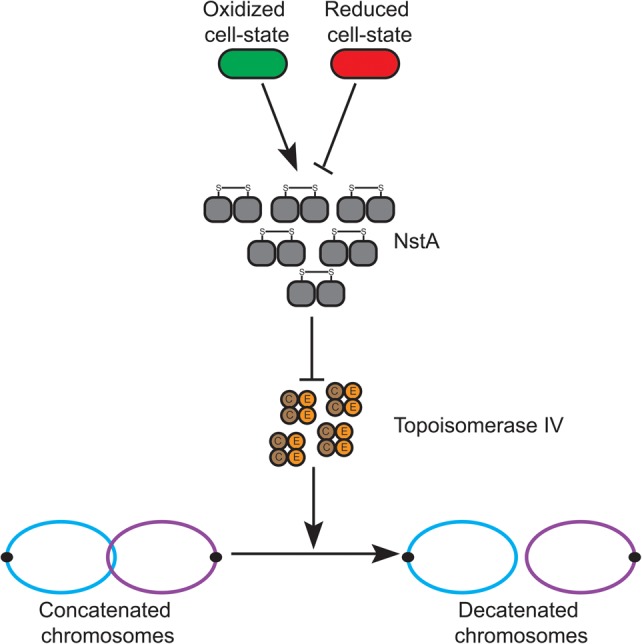
Model for NstA function. The activation of NstA (gray) by the formation of intermolecular cysteine disulfide bonds happens only when the cytoplasm is oxidized (green) and not in the reduced cell state (red). Upon activation, NstA inhibits the DNA decatenation activity of topo IV by specifically binding to the ParC (brown) subunit of the topo IV complex. The topo IV complex is a heterotetramer of ParC and ParE (orange).

Additionally, we demonstrated that, during the cell cycle, NstA is tightly regulated at the level of activity and abundance. We propose that the activity of NstA, dependent on the intermolecular cysteine disulfides, is regulated by the intracellular redox state of the cell (Fig. 6).

### Intracellular redox during cell cycle progression

One of the surprising revelations of our study is the dynamics of the intracellular redox rhythms during the bacterial cell cycle. Our results demonstrated that the cellular cytoplasm is in a reduced state in the G1 cells, with a short window of oxidation during G1 → S transition and early S phase, and regains the reduced state in the late stages of S phase (Fig. 5A). Our results also show that the intracellular redox state constitutes a non-protein-dependent activation mechanism for NstA to constrain topo IV activity temporally. The abundance of NstA and its activity through the intermolecular cysteine disulfide is presumably maximal while the cellular cytoplasm is in the oxidized state. The rhythmic changes of the intracellular redox state might well be a consequence of the metabolic state of the cell, and proteins such as NstA might be effectors linking metabolism with the cell cycle events. Such a function had been proposed earlier for the NADH-dependent cell division regulator KidO, which negatively regulates FtsZ, the tubulin homolog involved in cell division (Radhakrishnan et al. 2010). Nevertheless, the cycling of the NAD(P)H levels during the cell cycle, although proposed, remains to be demonstrated. The cell division regulatory role of KidO is confined to the G1 cells and the late S-phase cells (Radhakrishnan et al. 2010). Our results now demonstrate that the intracellular cytoplasm is in a reduced state in the G1-phase and late S-phase cells. This reduction of the cytoplasm might well be due to the increase in the pool of NAD(P)H in G1 phase and late S phase when KidO requires NADH to regulate cytokinesis.

A recent report had elegantly demonstrated that it is the G1 cells of the intracellular pathogen *Brucella abortus* that gain entry into the host cells during infection (Deghelt et al. 2014). More interestingly, the host-phagocytized G1 cells seem to recognize an undefined post-infection signal that triggers their entry into the S phase (Deghelt et al. 2014). In light of our discovery that the intracellular redox state changes during the cell cycle in *C. crescentus*, which shares many features with the *B. abortus* cell cycle (Hallez et al. 2004), it is tempting to speculate that the undefined post-infection signal is mediated by an intracellular redox change (caused by the oxidative attack of the host macrophages when they encounter *B. abortus*), which switches on the S-phase events and proliferation inside the host cell. Therefore, we propose that developing cells might be using dynamic intracellular redox rhythms as an activating mechanism to globally regulate the cell cycle. Such a mechanism might help the cells to fine-tune global regulatory cascades and, more importantly, coordinate developmental or infectious events with metabolism and/or environment. Such an idea is supported by several reports. Studies in human red blood corpuscles (RBCs) have shown that the rhythmic changes in NAD(P)H levels, which reflect the redox status, happen during circadian cycles of RBCs (O'Neill and Reddy 2011). Furthermore, these changes have been attributed to the transcription-independent regulation of molecular events during circadian cycles (O'Neill and Reddy 2011). Thus, we propose that the dynamics in the redox state not only is confined to the bacterial cell cycle but could well be used as a global cue to regulate developmental cycles in all other forms of life.

### Cell cycle regulation of topo IV activity

It is plausible that an oxidized environment along with the decatenation activity of topo IV might be fatal to the cells due to the nicking of DNA caused by both. Notwithstanding, there are other activities reported for topo IV in addition to decatenation that warrant its presence during early S phase; for example, the chromosome organization activity of ParC in association with the condensin subunit MukB demonstrated in *E. coli* (Hayama et al. 2013). The *C. crescentus* cells may overcome such a problem by using a negative regulator such as NstA, which specifically binds to ParC (Fig. 3E) and presumably prevents the interaction of ParC with ParE, whose function is stringently coupled to the oxidized environment, and whose presence is tightly regulated during the cell cycle at the level of transcription and abundance.

The transcriptional regulation of *nstA* production by GcrA and CcrM and the abundance at the post-translational level by the protease ClpXP ensure that the presence of NstA is temporally regulated, and its abundance is confined to the early to mid-S phase during the cell cycle (Fig. 1A, 2A). Overproduction of NstADD inhibits the cell cycle and chromosome segregation and induces toxicity in wild-type *C. crescentus* (Fig. 2F, 3A,B). The induction of toxicity by the cell cycle-stable NstADD is presumably due to the continued down-regulation of the topo IV decatenation activity even at the late stages of the chromosome cycle. The decatenation activity of topo IV is necessary, especially during the final stages of the chromosome cycle, to enable the proper segregation of the newly replicated chromosomes into the dividing daughter cells. Specific slowdown in the decatenation process alone, without any inhibition on further rounds of replication, leads to the production of multiple chromosomes and cell death, as seen in the NstADD-overproducing cells (Figs. 2F, 3B).

Our results show that the abundance of NstA during the cell cycle is regulated at the post-translational level by the protease ClpXP. Interestingly, ClpXP does not act on NstA at the same time point in the cell cycle as that seen for CtrA (Fig. 2A), KidO, or PdeA, which are known to be degraded by ClpXP during the G1 → S transition (Domian et al. 1997; Radhakrishnan et al. 2010; Abel et al. 2011). Rather, NstA is proteolized during the S → G2 time interval in a fashion similar to that recently reported for the divisome component FtsZ (Williams et al. 2014). The mechanism that regulates the switching of the activity of ClpXP during different time points in the cell cycle is an area of investigation for future. It is logical to think that this can be mediated by CpdR, PopA, and RcdA, the adaptors of ClpXP (Iniesta et al. 2006; McGrath et al. 2006; Duerig et al. 2009; Rood et al. 2012; Smith et al. 2014). Nevertheless, the activity of ClpXP is present during the initial and late S phase of the *C. crescentus* cell cycle, degrading substrates such as CtrA (Domian et al. 1997), SocB (Aakre et al. 2013), and NstA (this study), which regulate different stages of chromosome replication, elongation, and segregation. Therefore, we propose that ClpXP plays a major role in controlling the entire chromosome cycle.

## Materials and methods

### Growth conditions and media

*C. crescentus* strains were grown on rich PYE medium (0.2% peptone, 0.1% yeast extract, 1 mM MgSO_4_, 0.5 mM CaCl_2_) or minimal M2G medium (M2-1X salt solution [0.87 g/L Na_2_HPO_4_, 0.53 g/L KH_2_PO_4_, 0.25 g/L NH_4_Cl] supplemented with 0.5 mM MgSO_4_, 0.2% glucose, 10 µM FeSO_4_.EDTA, 0.5 mM CaCl_2_) (Ely 1991) and incubated at 29°C unless specifically mentioned. The *C. crescentus* strains were subjected to electroporation, synchronization, øCr30-mediated transductions, and intergeneric conjugations (using *E.coli S17-1*) as previously described (Poindexter 1964; Chen et al. 2005; Radhakrishnan et al. 2008). *E. coli* strains EC100D (Epicentre), S17-1, and XL-1 Red (Agilent Technologies) were grown on LB medium and incubated at 37°C unless specifically mentioned.

### Microscopy

Differential interference contrast (DIC) and fluorescence microscopy were performed on a Nikon Eclipse 90i microscope equipped with a 100× oil TIRF (1.49 numerical aperture) objective and a coolSNAP HQ-2 (Photometrics) CCD camera. Cells were placed on 1% agarose solidified pads for imaging. Images were processed and analyzed with Metamorph software (Molecular Devices).

### Flow cytometry analyses

Flow cytometry analyses were performed as described earlier (Murray et al. 2013). Cells were incubated until mid-log phase, and 1 mL of culture was transferred into 9 mL of ice-cold 70% ethanol and stored overnight at −20°C for fixation. Two milliliters of the fixed cells was washed with 1 mL of staining buffer (10 mM Tris-HCl at pH 7.20, 1 mM EDTA, 50 mM sodium citrate, 0.01% Triton-X-100). The cells were then harvested by centrifugation at 8000 rpm for 5 min, and the pellet was resuspended in 1 mL of staining buffer containing 0.1 mg/mL RNase A (Roche) and incubated for 30 min at room temperature. The cells were pelleted at 8000 rpm for 5 min, and the pellet was resuspended in 1 mL of staining buffer containing 0.5 μM SYTOX green nucleic acid stain (Molecular Probes). The cells were incubated in the dark for 5 min and analyzed using an Accuri C6 flow cytometer (BD Biosciences) equipped with an argon ion laser. Relative chromosome number was directly estimated from the green fluorescence (FL1-A) value of the stained cells and analyzed using BD Accuri C6 software.

### DNA decatenation assay

Decatenation assays using purified ParC, ParE, BSA, and wild-type and mutant NstA proteins were performed using the DNA topo IV assay kit (Profoldin) with slight modifications to the manufacturer's instructions. A 10× reaction buffer without DTT (200 mM Tris-HCl at pH 8.35, 350 mM ammonium acetate, 46% glycerol, 0.05% igepal, 80 mM magnesium chloride) was used instead of the reaction buffer supplied by the manufacturer. Purified *C. crescentus* ParC and ParE (0.01 μM each) and 2–8 μM wild-type NstA or 8 μM mutant NstA were used in a 50-μL reaction containing 1× reaction buffer, 2 μg/mL catenated DNA, and 1 mM ATP. The control reaction without ATP contained 8 μM NstA and 0.01 μM each ParC and ParE. An amount of BSA equivalent to 8 μM NstA was used. For the experiments involving DTT, 8 μM purified NstA was preincubated with 6 mM DTT for 30 min before the addition of the ParC, ParE, catenated DNA, and ATP components. The data represented are from three independent experiments. The SEM shown in the figures was derived with Origin 7.5 software (OriginLab Corporation). For visualization on gel, the reaction mixtures were analyzed on a 1.2% (w/v) agarose gel with 0.5× TBE running buffer (44.5 mM Tris, 44.5 mM boric acid, 1 mM EDTA). The gel was run at 25 V for 6 h and visualized by UV after staining with ethidium bromide.

### Protein stability analyses

Wild-type cells harboring either *xylX::*P*_xyl_-gfp-nstA* or *xylX::*P*_xyl_-gfp-nstADD* grown overnight in M2G were diluted into 25 mL of fresh M2G and were further incubated until 0.2 OD at 600 nm was attained. Xylose (0.3%) was added to induce the expression of *gfp-nstA* and *gfp-nstA*DD for 4 h. The cells were then treated with 1 µg/mL chloramphenicol. Samples (1.5 mL) were collected at 5-min intervals and harvested by centrifugation at 13,000 rpm for 1 min. Cell pellets collected at each time point were frozen on dry ice, and the lysates were analyzed by immunoblot.

To analyze the influence of ClpP and ClpX on the stability of GFP-NstA, strains Δ*nstA ΔsocB ΔclpP xylX::*P_*xyl*_-*gfp-nstA* + pP*_lac_- clpP* and Δ*nstA ΔsocB ΔclpX xylX::*P_*xyl*_-*gfp-nstA* + pP*_lac_-clpX* were grown in the presence or absence of 1 mM IPTG to induce or deplete the expression of ClpP and ClpX, respectively. Expression of *gfp-nstA* was induced with 0.3% xylose for 4 h prior to the addition of chloramphenicol. The abundance of GFP-NstA was monitored over time by immunoblot analyses of samples collected every 5 min after the addition of chloramphenicol.

### Analyses of intracellular redox

Wild-type cells harboring *xylX::*P*_xyl_-rogfp2* grown overnight in M2G were diluted into 50 mL of fresh M2G and further incubated until 0.2 OD at 600 nm was attained. The cells were then treated with 0.3% xylose for 3 h to induce the expression of *rogfp2.* The culture was then harvested by centrifugation at 8500 rpm for 5 min. The pellet was washed three times with ice-cold M2-1X salt solution by centrifugation at 7000 rpm for 3 min and finally resuspended in 700 µL of M2-1X salt solution followed by the addition of 750 µL of Percoll (GE Healthcare). The mixture was then subjected to centrifugation at 10,000 rpm for 15 min at 4°C. The swarmer band (bottom) was isolated and washed three times using ice-cold M2-1X salt solution by centrifugation at 7000 rpm for 3 min at 4°C. Finally, the swarmer pellet was resuspended in 2 mL of M2G supplemented with 0.3% xylose and incubated on a rocker kept at 29°C. One-hundred microliters of culture was taken at 10-min intervals, and fluorescence intensities at 405-nm and 488-nm excitation wavelengths were measured at every time point with emission at 510 nm using SpectraMax i5 (Molecular Devices). In addition to fluorescence intensities, OD at 660 nm was also taken every 10 min using the SpectraMax i5. Polystyrene flat-bottomed 96-well plates (NEST Biotechnologies Ltd.) were used to collect the readings. The data represented are from eight independent experiments, and the 405-/488-nm ratios were normalized with respect to OD at 660 nm at time point 0. The data were plotted, and SEM was derived using Origin 7.5 software (OriginLab Corporation).

For analyzing the functionality of roGFP2, freshly grown wild-type cells harboring *xylX::*P_*xyl*_-*rogfp2* were treated with either 2 mM H_2_O_2_ for 10 min or 20 mM DTT for 20 min after 3 h of treatment with xylose to induce the production of roGFP2. Fluorescence intensities at 405 nm and 488 nm and OD at 660 nm were monitored after DTT or H_2_O_2_ treatment along with untreated control samples. The data represented are from four independent experiments.

### Analyses of disulfide bridges

To analyse the disulfide linkage formation in NstA, we used the method described previously (Schmalen et al. 2014). Briefly, the mid-log cultures (100 mL) were induced with 0.3% xylose for 3 h and pelleted. The cell pellets were then washed three times with PBS buffer and resuspended in MOPS-G buffer (25 mM MOPS at pH 7.1, 5 mM EDTA, 150 mM NaCl, 0.1% igepal, 6 M guanidine HCl) containing 50 mM NEM. The resuspended cells were sonicated on ice using two bursts of 30 sec, and the crude extract was centrifuged to remove cellular debris. The supernatant was then transferred to a new reaction tube and incubated for 2 h at room temperature in order to facilitate the blocking of free thiol groups by NEM. To remove excess NEM, proteins were precipitated by addition of 4 vol of methanol and incubation overnight at −20°C. The precipitate was then collected by centrifugation and washed five times with methanol. The pellets were dried, dissolved in MOPS-G buffer, and equally distributed to four reaction tubes. Two tubes were left untreated as controls, the other tubes were treated with 20 mM DTT or 5 mM H_2_O_2_ , and all tubes were incubated for 1 h at room temperature. Excess DTT and H_2_O_2_ were removed by precipitating the proteins using 4 vol of methanol and incubated for 1 h at −20°C. The precipitates were then washed three times with methanol, dried, and finally resuspended in SEENS buffer (0.1 M sodium phosphate at pH 6.5, 10 mM EDTA, 0.1% nonidet P40, 3% SDS) containing 300 µM MP (Sigma-Aldrich) and incubated for 1 h at room temperature. One of the control samples was not treated with MP. The resuspended samples were then concentrated using Amicon centrifugal tubes (Millipore Corporation). The concentrated samples were resolved in an SDS–polyacrylamide gel with SDS gel loading buffer (50 mM Tris at pH 6.8, 10% glycerol, 2% SDS, 0.02% bromophenol blue). The samples were not boiled to avoid nonspecific reaction of MP with amino groups and were analyzed by immunoblot with monoclonal GFP antibodies.

Details of protein expression and purification, β-galactosidase assays, immunoprecipitations, immunoblots, suppressor screens, and strain and plasmid constructions are in the Supplemental Material.

## Supplementary Material

Supplemental Material
