# Supplementary material for: A cell cycle-controlled redox switch regulates the topoisomerase IV activity
Source: Genes Dev. 2015 Jun 1;29(11):1175–87. doi: 10.1101/gad.257030.114 (PMC4470285; doi:10.1101/gad.257030.114)
Supplement: Supplemental Material [file supp_29_11_1175__index.html]

Supplemental Material 

# A cell cycle-controlled redox switch regulates the topoisomerase IV activity

## Supplemental Material

**Files in this Data Supplement:**

- Supplemental Material.pdf
