# Supplementary material for: A cell cycle-controlled redox switch regulates the topoisomerase IV activity
Source: Genes Dev. 2015 Jun 1;29(11):1175–87. doi: 10.1101/gad.257030.114 (PMC4470285; doi:10.1101/gad.257030.114)
Supplement: Supplemental Material [file supp_29.11.1175_Supplemental_Material.pdf]

SUPPLEMENTAL INFORMATION

MATERIALS AND METHODS

Strain constructions

In-frame deletions of the sequence encoding residues 9-59 of NstA were created in strains NA1000 (*WT*), ML2031 (Δ*socB* Δ*clpP* + *p*P$_{lac}$-*clpP*) (Aakre et al. 2013) and ML2032 (Δ*socB* Δ*clpX* + *p*P$_{lac}$-*clpX*) (Aakre et al. 2013) using the standard two-step recombination sucrose counter-selection procedure induced by the *p*NPTS138-derived Δ*nstA* deletion plasmid, *p*SN123, yielding **SKR1797** (*ΔnstA*), **SN920** (*ΔnstA ΔclpP ΔsocB* + *p*P$_{lac}$-*clpP*) and **SN928** (*ΔnstA ΔclpX ΔsocB* + *p*P$_{lac}$-*clpX*) respectively.

NA1000 was transformed with *p*MT335 and derivatives *p*SKR114 (*p*MT335-*P$_{van}$-nstA*), *p*SKR126 (*p*MT335-*P$_{van}$-nstADD*), *p*SN8 [*p*MT335-P$_{van}$-*nstA*(C23R)DD], *p*SN10 [*p*MT335-P$_{van}$-*nstA*(C8Y)DD], *p*SN12 [*p*MT335-P$_{van}$-*nstA*(T5I)DD], *p*SN14 [*p*MT335-P$_{van}$-*nstA*(F7L)DD], *p*SN16 [*p*MT335-P$_{van}$-*nstA*(L36F)DD], *p*SN172 [*p*MT335-P$_{van}$-*nstA*(Q2R)DD], *p*SN173 [*p*MT335-P$_{van}$-*nstA*(E47K)DD], *p*SN174 [*p*MT335-P$_{van}$-*nstA*(V20M)DD], *p*SN175 [*p*MT335-P$_{van}$-*nstA*(D12G)DD] and *p*SN176 [*p*MT335-P$_{van}$-*nstA*(F56L)DD] to yield **SKR1783** (*WT* + *p*MT335), **SKR1779** (*WT* + *p*P$_{van}$-*nstA*), **SKR1800** (*WT* + *p*P$_{van}$-*nstADD*), **SN1113** (*WT* + *p*P$_{van}$-*nstA*(C23R)DD), **SN1114** (*WT* + *p*P$_{van}$-*nstA*(C8Y)DD), **SN1115** (*WT* + *p*P$_{van}$-*nstA*(T5I)DD), **SN170** (*WT* + *p*P$_{van}$-*nstA*(F7L)DD), **SN1117** (*WT* + *p*P$_{van}$-*nstA*(L36F)DD, **SN176** (*WT* + *p*P$_{van}$-*nstA*(Q2R)DD), **SN1116** (*WT* +

*p*P$_{van}$-*nstA*(E47K)DD), **SN175** (*WT* + *p*P$_{van}$-*nstA*(V20M)DD), **SN171** (*WT* + *p*P$_{van}$-*nstA*(D12G)DD) and **SN173** (*WT* + *p*P$_{van}$-*nstA*(F56L)DD), respectively.

Strains **SN47** (*WT xylX::*P$_{xyl}$-*nstA*), **SN278** (*WT xylX::p*XGFP4) and **SN489** (*WT xylX::*P$_{xyl}$-*nstA*DD) were made by electroporating NA1000 with *p*SN83 (*p*XGFP4-P$_{xyl}$-*nstA*), pXGFP4 vector, and *p*SN87 (*p*XGFP4-P$_{xyl}$-*nstA*DD), respectively.

Strains **SN258** (WT *xylX::*P$_{xyl}$-*nstA*(C8Y)-*gfp*), **SN260** (WT *xylX::*P$_{xyl}$-*nstA*-*gfp*) and **SN264** (WT *xylX::*P$_{xyl}$-*nstA*(C23R)-*gfp*) were made by electroporating the plasmids *p*SN195 (*p*XGFP4-P$_{xyl}$-*nstA*(C8Y)-*gfp*), *p*SN36(*p*XGFP4-P$_{xyl}$-*nstA*-*gfp*) and *p*SN40(*p*XGFP4-P$_{xyl}$-*nstA*(C23R)-*gfp*) into NA1000, respectively.

NA1000, JC948 (Δ*ccrM ftsZ P$_{xyl}$-ftsZ*) (Gonzalez and Collier 2013), LS3707 (Δ*gcrA xylX::*P$_{xyl}$-*gcrA*) *(Holtzendorff et al. 2004)* and YB1585 (*ftsZ P$_{xyl}$-ftsZ)* (Wang et al. 2001) strains were transformed with *p*SN59 (*p*lac290-P$_{nstA}$-*lacZ*) to yield **SN331** (*WT* + *p*lac290-P$_{nstA}$-*lacZ*), **SN551** (Δ*ccrM ftsZ P$_{xyl}$-ftsZ* + *p*lac290-P$_{nstA}$-*lacZ*), **SN1110** (Δ*gcrA xylX::*P$_{xyl}$-*gcrA* + *p*lac290-P$_{nstA}$-*lacZ*) and SN618 (*ftsZ P$_{xyl}$-ftsZ* + *p*lac290-P$_{nstA}$-*lacZ*), respectively.

Strain **SN481** (*WT hu::hu-cfp* + *p*P$_{van}$-*nstA*) was made by electroporating *p*SKR114 into MT42 (*WT hu::hu-cfp*). MT42 was a gift from Martin Thanbichler.

Strains **SN519** (*WT xylX::*P_{xyl}-*gfp-nstA*) and **SN521** (*ΔnstA xylX::*P_{xyl}- *gfp-nstA)* were made by electroporating the plasmid *p*SN160 (*p*XGFP4-C1-P_{xyl}-*gfp-nstA)* into NA1000 and SKR1797 respectively.

Strains **SN638** (*WT xylX::pXGFP4*-C1) and **SN624** (*WT xylX::*P_{xyl}-*gfp-nstADD)* were generated by electroporating the plasmids *p*XGFP4-C1 and *p*SN113 (*p*XGFP4-C1-P_{xyl}-*gfp-nstADD*), respectively, into NA1000.

Strains **SN648** (*xylX::*P_{xyl}-*parC-gfp* + *p*MT335), **SN650** (*xylX::*P_{xyl}-*parC-gfp* + *p*P_{van}-*nstA*-TAP*)*, **SN670** (*xylX::*P_{xyl}-*parC-gfp* + *p*P_{van}-*nstA*(C23R)- TAP), and **SN672** (*xylX::*P_{xyl}-*parC-gfp* + *p*P_{van}-*nstA*(V20M)-TAP) were made by transforming the strain LS3744 (*WT xylX::*P_{xyl}-*parC-gfp*) (Wang and Shapiro 2004) with *p*MT335, *p*SN94 (*p*MT335-P_{van}-*nstA*-TAP)*, p*SN129 (*p*MT335-P_{van}-*nstA*(C23R)-TAP) and *p*SN132 (*p*MT335-P_{van}-*nstA*(V20M)-TAP), respectively.

Strains **SN652** (*xylX::*P_{xyl}-*gfp-ParE* + *p*MT335), **SN654** (*xylX::*P_{xyl}-*gfp-ParE* + *p*P_{van}-*nstA*-TAP*)*, **SN674** (*xylX::*P_{xyl}-*gfp-ParE* + *p*P_{van}-*nstA*(C23R)-TAP) and **SN676** (*xylX::*P_{xyl}-*gfp-ParE* + *p*P_{van}-*nstA*(V20M)-TAP) were made by transforming the strain LS3745 (*WT xylX::*P_{xyl}-*gfp-parE*) (Wang and Shapiro 2004) with *p*MT335, *p*SN94 (*p*MT335-P_{van}-*nstA*-TAP)*, p*SN129 (*p*MT335-P_{van}-*nstA*(C23R)-TAP) and *p*SN132 (*p*MT335-P_{van}-*nstA*(V20M)-TAP), respectively.

Strain **SN950** (*ΔnstA* + *p*MT335-P_{van}-*nstA*DD) was made by electroporating the plasmid *p*SKR126 into SKR1797.

Strains **SN966** (*ΔclpP ΔsocB xylX::*P_{xyl}-*gfp-nstA* + *p*P_{lac}-*clpP*) and SN970 (*ΔclpX ΔsocB xylX::*P_{xyl}-*gfp-nstA* + *p*P_{lac}-*clpX*) were made electroporating *p*SN160 into ML2031 and ML2032 respectively.

Strain **SN1111** (*WT hu::hu-cfp* + *p*P_{van}-*nstADD*) was made by electroporating *p*SKR126 into MT42 (*WT hu::hu-cfp*). MT42 was a gift from Martin Thanbichler.

Strain **SN1017** (CB15 *xylX::*P_{xyl}-*nstADD*) was made by electroporating *p*SN87 into CB15.

Strains **SN1021** (*parE*ts *xylX::p*XGFP4) and **SN1023** (*parE*ts *xylX::*P_{xyl}-*nstADD*) were made by electroporating *p*XGFP4 and *p*SN87, respectively, into *parE*ts mutant, PC8830 (Ward and Newton 1997).

Strains **SN1027** (*parC*ts *xylX::p*XGFP4) and **SN1029** (*parC*ts *xylX::*P_{xyl}-*nstADD*) were made by electroporating *p*XGFP4 and *p*SN87, respectively, into *parC*ts mutant, PC8861 (Ward and Newton 1997).

Strains **SKR1809** (*WT* + *p*P_{van}*-nstA-segfp*), **SN1118** (*WT* + *p*P_{van}-*nstA*(C8Y)-*segfp*), **SN1119** (*WT* + *p*P_{van}*-nstA*(C23R)-*segfp*) and **SN1120** (*WT* + *p*P_{van}*-nstA*(V20M)-*segfp*) were made by electroporating the plasmids *p*MT335, *p*SKR131 (*p*MT335-P_{van}*-nstA-segfp),* *p*SN177 (*p*MT335-P_{van}*-nstA*(C8Y)-*segfp),* *p*SN178 (*p*MT335-P_{van}*-nstA(C23R)-segfp*) and *p*SN179 (*p*MT335-P_{van}-*nstA*(V20M)-*segfp*), respectively, into NA1000.

Strain **SN1121** (*WT xylX::*P_{xyl}*-rogfp2*) was generated by electroporating *p*SKR218 (*p*XGFP4-P_{xyl}-*rogfp2*)into NA1000.

Strains **SN1161** (*WT* + *p*P_{van}-*gfp-nstA*) and **SN1163** (*WT* + *p*P_{van}-*gfp-nstA*DD) were made by electroporating *p*SKR273 (*p*MT335-P_{van}-*gfp-nstA*) and *p*SKR274 (*p*MT335-P_{van}-*gfp-nstA*DD) into NA1000, respectively.

Strain **SN1165** [*WT* + *p*P_{van}-*nstA*(C23S)DD] was made by electroporating *p*SN250 (*p*MT335-P_{van}-*nstA*(C23S)DD) into NA1000.

Strains **SN1176** (*WT nstA*::P_{nstA}-*nstA*DD) and **SN1178** (WT *nstA*::P_{nstA}-*nstA-gfp*) were made by electroporating *p*SN242 (*p*GFP-C2-P_{nstA}-*nstADD*) and *p*SN244 (*p*GFP-C2-P_{nstA}-*nstA-gfp*) into NA1000.

Strains **SN1185** (*ΔnstA ΔsocB ΔclpP xylX::*P_{xyl}-*gfp-nstA* + *p*P_{lac}-*clpP)* and **SN1186** (*ΔnstA ΔsocB ΔclpX xylX::*P_{xyl}-*gfp-nstA* + *p*P_{lac}-*clpX)* were made by

electroporating *p*SN160 (*p*XGFP4-C1-P$_{xyl}$-*gfp-nstA*) into SN920 and SN928, respectively.

Plasmid Constructions

NstA (CCNA_3091) is encoded from nucleotide positions 3240500-3240700 relative to the NA1000 genome sequence (CP001340). All constructions listed below are based on these coordinates. To allow proper placement of *nstA* relative to the ribosome binding sites of *vanA* or *xylX,* the ATG start codon carried an overlapping *Nde*I recognition sequence. The *nstA* constructs were made by cleavage with *Nde*I and *Eco*RI, an additional site appended to the stop codon at *nstA* 3' end, unless specifically mentioned.

Plasmid ***p*SKR114** (*p*MT335-P$_{van}$-*nstA*) was made by ligating the *nstA* allele (generated by PCR) as *Nde*I/*Eco*RI fragment into *p*MT335 (Thanbichler et al. 2007) that had been digested with the same restriction enzymes.

Plasmid ***p*SKR126** (*p*MT335-P$_{van}$-*nstA*DD*)* was made by PCR amplifying *nstADD* (in which the C-terminal Ala-Ala codons were replaced with Asp-Asp (GAU, GAC) codons) and cleaving the fragment with *Nde*I and *Eco*RI and ligating into *Nde*I/*Eco*RI treated *p*MT335.

To obtain intragenic suppressors of *nstA*DD overexpression, we sought spontaneous point mutants in *nstADD* by transforming *E.coli* XL1-Red

(Stratagene) mutator strain with *p*SKR126, thereby generating the plasmids ***p*SN8** (*p*MT335-P$_{van}$-*nstA*(C23R)DD), ***p*SN10** (*p*MT335-P$_{van}$-*nstA*(C8Y)DD), ***p*SN12** (*p*MT335-P$_{van}$-*nstA*(T5I)DD), ***p*SN14** (*p*MT335-P$_{van}$-*nstA*(F7L)DD), ***p*SN16** (*p*MT335-P$_{van}$-*nstA*(L36F)DD), ***p*SN172** (*p*MT335-P$_{van}$-*nstA*(Q2R)DD), ***p*SN173** (*p*MT335-P$_{van}$-*nstA*(E47K)DD), ***p*SN174** (*p*MT335-P$_{van}$-*nstA*(V20M)DD), ***p*SN175** (*p*MT335-P$_{van}$-*nstA*(D12G)DD) and ***p*SN176** (*p*MT335-P$_{van}$-*nstA*(F56L)DD).

To make plasmids ***p*SN36** (*p*XGFP4-P$_{xyl}$-*nstA*-*gfp*), ***p*SN40** (*p*XGFP4-P$_{xyl}$-*nstA*(C23R)-*gfp*) and ***p*SN195** (*p*XGFP4-P$_{xyl}$-*nstA*(C8Y)-*gfp*), *nstA* alleles were generated by PCR such that the stop codon was substituted with an *Eco*RI site in the same reading frame. The mutant alleles were then ligated upstream of *gfp* in *p*XGFP4 as *NdeI*/*Eco*RI fragments.

To make the P$_{nstA}$-*lacZ* transcriptional reporter *p*lac290-P$_{nstA}$*lacZ,* nucleotides 3240210-3240589 of NA1000 genome (CP001340) was amplified and ligated as *Eco*RI/*Hind*III fragment into the plasmid *p*lac290 to drive the transcription of the promoterless *lacZ* gene.The resulting plasmid was named as ***p*SN59**.

The plasmids ***p*SN83** (*p*XGFP4-P$_{xyl}$-*nstA*) and ***p*SN87** (*p*XGFP4-P$_{xyl}$-*nstA*DD) were made by releasing the *nstA* and *nstADD* fragments from *p*SKR114 and *p*SKR126, respectively, after cleavage with *Nde*I/*Eco*RI enzymes and

ligating into *p*XGFP4 vector (M.R.K Alley unpublished) digested with the same restriction enzymes.

The plasmids for NstA-TAP and its mutant derivatives were made from *p*CWR589 (Radhakrishnan et al. 2010) by replacing *kidO* fragment with *nstA* wild type or mutant alleles using *Nde*I/*Eco*RI restriction enzymes. The *nstA* alleles were generated by PCR such that the stop codon was substituted with an *Eco*RI site in the same reading frame. The resulting plasmids were named as ***p*SN94** (*p*MT335-P_{van}-*nstA*-TAP), ***p*SN129** (*p*MT335-P_{van}-*nstA*(C23R)-TAP) and ***p*SN132** (*p*MT335-P_{van}-*nstA*(V20M)-TAP).

The *E.coli* overexpression plasmids for His_6-SUMO-NstA and its mutant derivatives were made from pCWR551 (Radhakrishnan et al. 2010) by replacing *ftsZ* fragment with *nstA* wild type or mutant alleles using *Nde*I/*Sac*I restriction enzymes. The resulting plasmids were named ***p*SN106** (His_6-SUMO-*NstA*), ***p*SN141** [His_6-SUMO-NstA(C23R)] and ***p*SN144** [His_6-SUMO-*NstA*(V20M)].

Plasmids ***p*SN113** (*p*XGFP4-C1-P_{xyl}-*gfp-nstA*DD) and ***p*SN160** (*p*XGFP4-C1-P_{xyl}-*gfp-nstA*) were made by PCR amplifying *nstA*DD and *nstA* alleles and cleaving them with *Bgl*II/*Eco*RI, wherein the predicted the start codon ATG was replaced with GTG that carried an overlapping *Bgl*II recognition site to allow proper placement of *nstA* facilitating N-terminal GFP fusion. The alleles were ligated into *p*XGFP4-C1 vector (M.R.K Alley unpublished) cut with *Bgl*II/*Eco*RI.

To make the *nstA* knock out plasmid ***p*SN123**, two 600bp (approx.) fragments extending from codon 7 beyond the 5' end and from codon 56 beyond the 3' end of *nstA* were amplified. The fragments were flanked by recognition sequences for EcoRI or BamHI at the 5' end and BamHI or HindIII at the 3' end respectively. After cleavage using these restriction enzymes, the fragments were joined by way of a three way ligation into pNPTS138 (M.R.K Alley, unpublished) that had been digested with EcoRI/HindIII via a medial BamHI fusion that was positioned in-frame.

The *E.coli* overexpression plasmid for His_6-SUMO-ParC was made using pCWR551 by replacing the *ftsZ* fragment with PCR amplified wild type *parC* allele using *Nde*I/*Sac*I restriction enzymes. The resulting plasmid was named ***p*SN137** (His_6-SUMO-*parC*).

The *E.coli* overexpression plasmid for His_6-SUMO-ParE was made from pCWR551 by replacing the *ftsZ* fragment with PCR amplified wild type *parE* allele using *Nde*I/*Xho*I restriction enzymes. The resulting plasmid was named ***p*SN142** (His_6-SUMO-*parE*).

The plasmids ***p*SKR131** (*p*MT335-P_{van}-*nstA*-*segfp*), ***p*SN177** (*p*MT335-P_{van}-*nstA*(C8Y)-*segfp*), ***p*SN178** (*p*MT335-P_{van}-*nstA*(C23R)-*segfp*) and *p*SN179 (*p*MT335-P_{van}-*nstA*(V20M)-*segfp*) were made by triple ligating *Nde*I/*Eco*RI digested PCR fragments of wild type *nstA* , *nstA*(C8Y), *nstA*(C23R) and

nstA(V20M) alleles, without the stop codon, respectively, along with *Eco*RI/*Xba*I prepared *segfp* (Pedelacq et al. 2006) fragment into *p*MT335 cut with *Nde*I and *Xba*I.

For making the plasmid ***p*SKR218** (*p*XGFP4-P$_{xyl}$-*rogfp2*), the *rogfp2* was PCR amplified from *p*QE-*GrX1-rogfp2* (Gutscher et al. 2008). The PCR amplified *rogfp2* was cleaved with *Eco*RI/*Xba*I and cloned into *p*XGFP4 cut with the same set of restriction enzymes. The resulting vector had the *egfp* of *p*XGFP4 replaced with *rogfp2* downstream of the xylose inducible (P$_{xyl}$) promoter.

Plasmid ***p*SKR273** (*p*MT335-P$_{van}$-*gfp-nstA*) was made by releasing the *gfp-nstA* from *p*SN160 (*p*XGFP4-C1-P$_{xyl}$-*gfp-nstA*) by EcoRI and *Nde*I and cloned into *p*MT335 digested with the same set of enzymes.

Plasmid ***p*SKR274** (*p*MT335-P$_{van}$-*gfp-nstA*DD) was made by releasing the *gfp-nstADD* from *p*SN113 (*p*XGFP4-C1-P$_{\mathrm{xyl}}$-*gfp-nstADD*) *EcoR*I and *Nde*I and cloned into *p*MT335 digested with the same set of enzymes.

Plasmid ***p*SN242** (*p*GFP-C2-P$_{\mathrm{nstA}}$-*nstADD*) was made by amplifying the nucleotide region 3239813-3240701 of NA1000 genome and ligating as *Nde*I/*Sac*I fragment into *p*GFPC-2 vector (Thanbichler et al. 2007). The C-terminal Ala-Ala codons of *nstA* were replaced with two Asp (GAU, GAC) codons in the primer used.

Plasmid ***p*SN244** (*p*GFP-C2-P$_{nstA}$-*nstA-gfp*) was made by amplifying the nucleotide region 3239813-3240701 of NA1000 genome and ligating as *Nde*I/*Sac*I fragment into *p*GFPC-2 vector (Thanbichler et al. 2007). The stop codon of *nstA* was substituted with SacI restriction site to read into the downstream *gfp* in the same reading frame.

Plasmid ***p*SN223** was constructed by two Single Primer Reactions IN Parallel (SPRINP) method (Edelheit et al. 2009) using Expand Long Template DNA Polymerase (Roche, IN). The plasmid *p*SN196 (*p*OK-*nstA*DD) was used as the template DNA for the reactions and the primers were made by replacing the Cys codon at 23rd position of *nstA* with Ser codon (TCC). The SPRINP reactions were then combined, denatured at 95 °C and gradually cooled for promoting random annealing of the parental DNA and the newly synthesized strands. The products were digested with *Dpn*I and then transformed into *E. coli*. The plasmids were then isolated from the transformants and analyzed by Sanger sequencing for confirming the point mutation. The clone *p*SN223 with the specific point mutation was further chosen.

Plasmid ***p*SN250** [*p*MT335-P$_{van}$-*nstA*(C23S)DD] was made releasing the *nstA(C23S)DD* fragment from *p*SN223 by *Nde*I/*EcoR*I and ligating into *p*MT335 cut with *Nde*I and *EcoR*I.

DAPI staining

For DAPI staining, 1 mL of bacterial culture was harvested by centrifugation at 4500g for 2 min. The pellets were then washed thrice with 1 mL of 1X-PBS (phosphate buffered saline [pH 7.40]). The pellets were then resuspended in 1 mL of 1X-PBS containing 10 µg/mL DAPI (Roche, Switzerland) followed by incubation in dark for 5 min. The cells were then harvested by centrifugation, and excess DAPI was removed by washing the pellet thrice with 1X-PBS. Finally the pellet was resuspended in 1X-PBS before imaging.

Re-synchronization

Mid-log phase cells, 1L, of the strain *WT xylX::gfp-nstA* grown in M2G was induced with 0.3% Xylose for 3 h, and subjected to large scale density gradient synchronization as described earlier (Radhakrishnan et al. 2010). The swarmer cells collected were further allowed to progress in cell cycle, for 140 min, by re-suspending in fresh M2G supplemented with 0.3% xylose. The cells at 140 min were again synchronized by the density gradient method to isolate newly formed swarmer and stalked cells. Lysates of these swarmer and stalked cells were then analyzed by immunobloting.

Non-reducing SDS-PAGE

The cell pellets were re-suspended in non-reducing SDS buffer (50 mM Tris [pH 6.8], 10 % Glycerol, 2% SDS, 0.02% bromophenol blue). Samples were heated

at 95°C for 3 min and centrifuged. Then the supernatant was used for SDS-PAGE followed by western blot analyses.

Immunoblots and Far Western analyses

Proteins from cell lysates were allowed to migrate on a polyacrylamide gel. Subsequently the proteins were transferred onto poly vinylidene fluoride membrane (PVDF, Millipore), at 4°C, by applying constant voltage. The blots were blocked for 1 h at room temperature with TBST solution (20 mM Tris-HCl [pH 7.5], 150 mM NaCl, 0.5% Tween-20, 5% non fat dry milk), followed by 1 h incubation in TBST solution with primary antibody of appropriate dilution (see below). The blots were then washed four times with TBS solution (20 mM Tris-HCl [pH 7.5], 150 mM NaCl), and detected with donkey anti-rabbit / anti-mouse secondary antibody conjugated to horseradish peroxidise (Jackson ImmunoResearch, PA, USA).

For Far Western analysis, the blots containing BSA, purified ParC, and ParE proteins (each 0.1 nM) were blocked in TBST solution for one hour and then incubated for 3 h at room temperature in TBST solution with 25 nM purified His_6-SUMO-NstA. The blot was washed four times with TBS solution, probed with monoclonal anti-His_6 antibody, and detected with donkey anti-mouse antibody conjugated to horseradish peroxidase (Jackson ImmunoResearch, PA, USA).

For the non-reducing SDS-PAGE experiments, cell pellets were re-suspended in non-reducing SDS buffer (50 mM Tris [pH 6.8], 10% Glycerol, 2% SDS, 0.02 % bromophenol blue). Samples were heated at 95°C for 3 min and centrifuged at 10,000rpm for 10 min to remove cell debris. The supernatant was then used for SDS-PAGE followed by immunoblot analyses.

The primary antibodies of the following dilutions were used: α-CtrA (Domian et al. 1997) - 1:15,000; α-GFP (Living Colors JL-8, Clontech Laboratories, CA, USA) - 1:20,000; α-His$_6$ (Cell Signaling, MA, USA) - 1:20,000; α-MreB (Figge et al. 2004) -1:40,000; α-MipZ (Thanbichler and Shapiro 2006)-1:10,000.

Cell cycle analyses of disulfide bound NstA-GFP

To analyze disulfide bound NstA-GFP during cell cycle, mid-log phase cells (2 L) of *WT xylX::*P$_{xyl}$-*nstA-gfp* grown in M2G, and induced with 0.3% xylose for 3 h, was subjected to large scale density gradient synchronization to isolate the swarmer cells. The isolated swarmer cells were then re-suspended in fresh M2G supplemented with 0.3% xylose, and samples were collected immediately after re-suspension (0 min), and at 60 min post re-suspension. The samples were then analyzed by non-reducing SDS-PAGE or analyzed for disulfide bridges by Mal-PEG (see main materials and methods) (Schmalen et al. 2014).

Protein expression and purification

Protein purification was done as described earlier (Radhakrishnan et al. 2010). Briefly, ParC, ParE, WT and mutant NstA proteins with an N-terminal, UlpI-cleavable His_6-SUMO (pSN106, pSN141, pSN144, pSN137, and pSN142) was expressed in *E. coli* Rosetta(DE3)/pLysS cells. The cells were then harvested by centrifugation, and proteins were purified under native conditions using Ni^{2+}-NitriloTriAcetate resin (Ni-NTA; Qiagen, Germany). After the UlpI cleavage of the His_6-SUMO tag (Bendezu et al. 2009), the purified proteins were used for DNA decatenation assays. 500 μM isopropyl-β-D-thiogalactoside (IPTG) was used to overexpress wild type His_6-SUMO-NstA and its mutant derivatives. 100 μM IPTG was used for overexpression of His_6-SUMO-ParC and His_6-SUMO-ParE.

β-Galactosidase assay

The cultures harbouring the P_{nstA}-*lacZ* (promoter of *nstA* transcriptionally fused to the *lacZ* reporter, pSN59) were incubated at 29°C till it reached 0.1-0.4 OD@660 nm (A_{660}). 50 μL of the cells were treated with a 10 μL of chloroform followed by the addition of 750 μL of Z-buffer (60 mM Na_2HPO_4, 40 mM NaH_2PO_4, 10 mM KCl, 1 mM $MgSO_4.7H_2O$, pH 7.0) followed by 200 μL of Ortho Nitro Phenyl-β-D-Galactoside (from stock concentration of 4 mg/mL dissolved in 100 mM potassium phosphate buffer [pH 7.0]). The reaction mixture was incubated at 30°C till yellow color was developed. Finally 500 μL of 1 M Na_2CO_3 solution was added to stop the reaction and absorbance at 420 nm (A_{420}) of the supernatant was noted using Z-buffer as the blank. The miller units (U) were calculated using

the equation U= (A_{420} X 1000) / (A_{660} X t X v), where ‘t’ is the incubation time (min), ‘v’ is the volume of culture taken (mL). For GcrA depletion experiments, *WT* Δ*gcrA*::Ω *xylX*::P_{xyl}-*gcrA* (Holtzendorff et al. 2004) was grown overnight in PYE supplemented with 0.3% xylose. The cells were harvested and washed 3 times with PYE, and then diluted appropriately into PYE supplemented with 0.3% xylose or PYE supplemented with 0.2% glucose for 5 h or 9 h. Experimental values were average of three independent experiments. The SEM shown in the figures was derived with Origin 7.5 software (OriginLab Corporation, Northhampton, MA, USA).

Tandem affinity purification(TAP)

Tandem affinity purification experiments were carried out as previously described (Radhakrishnan et al. 2010). Briefly, mid log phase cells (1 L) were induced with 50 mM vanillate and 0.3% Xylose for 3 h, harvested by centrifugation at 8500rpm for 10 min, washed in buffer I (50 mM sodium phosphate [pH7.4], 50 mM NaCl, 1 mM EDTA), and lysed at room temperature for 15 min in buffer II [(buffer I plus 10 mM $MgCl_2$, 0.5% n-dodecyl-β -D-maltoside (Pierce, IL, USA), 1x protease inhibitors (Complete^{TM} EDTA-free, Roche, Switzerland)] containing 50000 U of Ready-Lyse (Epicentre Technologies, WI, USA), and sonicated until the viscosity of the lysate was reduced. Cellular debris were removed by centrifugation at 10,000rpm for 10 min at 4°C. The supernatant was incubated with IgG Sepharose beads (GE Healthcare Bio-Sciences, Sweden) for 2 h at 4°C, washed thrice with IPP150 buffer (10 mM Tris-HCl [pH 8.0], 150 mM NaCl,

0.1% NP-40) and once with TEV cleavage buffer (IPP150 buffer plus 0.5 mM EDTA, 1 mM DTT), and incubated overnight at 4°C with TEV cleavage buffer containing 100 U/mL TEV protease (Promega, USA) to release the tagged complex. 3 μM $CaCl_2$ was added to the mix and incubated with Calmodulin Sepahrose beads (GE Healthcare Bio-Sciences, Sweden) for 1 h. The beads were then washed thrice with calmodulin binding buffer (10 mM β-mercaptoethanol, 10 mM Tris-HCl [pH 8.0], 150 mM NaCl, 1 mM magnesium acetate, 1 mM imidazole, 2 mM $CaCl_2$, 0.1% NP-40). The bound proteins were eluted using calmodulin elution buffer (calmodulin binding buffer substituted with 2 mM EGTA instead of 2 mM $CaCl_2$). The eluate was then concentrated using Amicon Centrifugal tubes (Millipore) and analyzed by immunoblot.

Quantitative chromatin immunoprecipitation (qChIP)

qChIP experiments were carried out as described earlier (Radhakrishnan et al. 2008). Mid-log phase cells were cross-linked in 10 mM sodium phosphate (pH 7.6) and 1% formaldehyde at room temperature for 10 min and on ice for 30 min thereafter, washed thrice in phosphate buffered saline (pH 7.4) and lysed in 5000 U of Ready-Lyse lysozyme solution (Epicentre Technologies, WI, USA). Lysates were sonicated on ice using 7 bursts of 30 sec to shear DNA fragments to an average length of 0.3-0.5 kbp. The cell debris were cleared by centrifugation at 14,000rpm for 2 min at 4°C. Lysates were normalized by protein content, diluted to 1 mL using ChIP buffer (0.01% SDS, 1.1% Triton X-100, 1.2 mM EDTA, 16.7 mM Tris-HCl [pH 8.1], 167 mM NaCl plus protease inhibitors [Complete^{TM} EDTA-

free, Roche, Switzerland]), and pre-cleared with 80 µL of protein-A agarose (Roche, Switzerland) saturated with 100 µg BSA and 300 µg Salmon sperm DNA. Ten% of the supernatant was removed and used as total chromatin input DNA. To the remaining supernatant anti-GcrA (Holtzendorff et al. 2004) or anti-m6A (Synaptic Systems, Germany) antibody was added (1:500 dilution), and incubated overnight at 4°C. Immuno complexes were trapped with 80 µL of protein-A agarose beads pre-saturated with BSA-salmon sperm DNA. The beads were then washed once each with low salt buffer (0.1% SDS, 1% Triton X-100, 2 mM EDTA, 20 mM Tris-HCl [pH 8.1], 150 mM NaCl), high salt buffer (0.1% SDS, 1% Triton X-100, 2 mM EDTA, 20 mM Tris-HCl [pH 8.1], 500 mM NaCl) and LiCl buffer (25 mM LiCl, 1% NP-40, 1% sodium deoxycholate, 1 mM EDTA, 10 mM Tris-HCl [pH 8.1]), and twice with TE buffer (10 mM Tris-HCl [pH 8.1], 1 mM EDTA). The protein•DNA complexes were eluted in 500 µL freshly prepared elution buffer (1% SDS, 1 mM $NaHCO_3$), supplemented with NaCl to a final concentration of 300 mM and incubated overnight at 65°C to reverse the crosslinks. The samples were treated with 2 µg of Proteinase K (Roche, Switzerland) for 2 h at 45°C in 40 mM EDTA and 40 mM Tris-HCl (pH 6.5). DNA was extracted using phenol:chloroform:isoamyl alcohol (25:24:1), ethanol-precipitated using 20 µg of glycogen as carrier, and resuspended in 50 µL of sterile deionized water. For GcrA depletion experiments the cells were treated as described above.

Quantitative PCR (qPCR) analyses

qPCR was performed on a CFX96 Real Time PCR System (Bio-Rad, CA, USA) using 10% of each ChIP sample, 12.5 µL of SYBR® green PCR master mix (Bio-Rad, CA, USA), 200 nM of primers and 6.5 µL of water per reaction. Standard curve generated from the cycle threshold (Ct) value of the serially diluted chromatin input was used to calculate the % input value of each sample. Average values are from triplicate measurements done per culture. The final data was generated from three independent cultures. The SEM shown in the figures was derived with Origin 7.5 software (OriginLab Corporation, Northhampton, MA, USA). The DNA region analysed after immunoprecipitation with anti-GcrA or anti-m6A antibody was from -279 to +27 relative to the start codon of *nstA*.

Intragenic suppressor screen

To screen for intragenic suppressor mutations in *nstA*DD, we made use of *E. coli* mutator strain XL1-Red (Stratagene). The plasmid *p*MT335-P$_{van}$-*nstA*DD was transformed into XL1-Red strain, and the mutant plasmid library obtained was then electroporated into *WT C. crescentus* cells. The toxicity minus transformants were selected by screening for growth in the presence of vanillate (note that wild type *nstADD* causes toxicity in the presence of vanillate). Further, the plasmids were isolated from individual colonies and then the *nstA*DD fragment was sub-cloned into fresh *p*MT335 vector digested with *Eco*RI/*Nde*I. The sub-cloned plasmids were transformed into fresh *WT* cells to make sure that the toxicity is

due to a mutation carried on the plasmid. Sanger sequencing of plasmids was done to identify the mutation on *nstA*.

Analyses of the effect of NEM on chromosome replication

To analyze the effect of NEM during the cell cycle, *WT* cells harboring *p*MT335 or *p*MT335-P$_{van}$-*nstA*(C23R)DD were grown overnight and were diluted into fresh PYE media containing 0.5 mM vanillate to induce the expression of *nstA*(C23R)DD. The culture was then treated with 7.5 µM or 10 µM of NEM prior to synchronization, and synchronized by percoll-gradient centrifugation, as described above, to isolate the G1 or swarmer cells. Finally the swarmer pellet was re-suspended in 12 mL PYE supplemented with 0.5 mM vanillate and 7.5 µM or 10 µM NEM and incubated on a shaker at 29°C. For the NEM untreated control, the above procedure was carried out in the absence of NEM. Samples were collected at every 20 min and analyzed by flow cytometry as described above.

SUPPLEMENTAL FIGURES

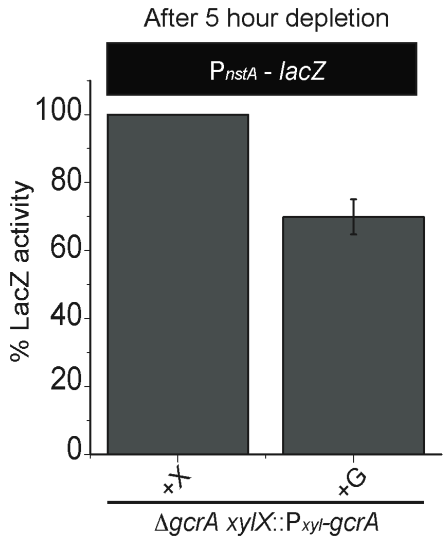

Figure S1. GcrA dependent regulation of P_{nstA}. Data of β-galactosidase assay using P_{nstA}-*lacZ* reporter plasmid to determine the *nstA* promoter activity in the presence and absence of *gcrA*. The values, ±SE, are from three independent experiments. The cells were grown in the presence of xylose (+X) to induce the production of GcrA, or in glucose (+G) to deplete GcrA for 5 h.

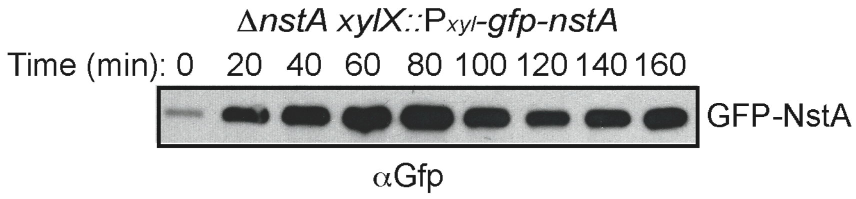

Figure S2. Cell cycle abundance of NstA. Cell cycle immunoblot analysis of GFP-NstA in synchronized population of Δ*nstA* cells harboring *gfp-nstA* at the *xylX* locus on the chromosome (*xylX*::P_{xyl}-*gfp-nstA*). The expression of *gfp-nstA* was induced with 0.3% xylose.

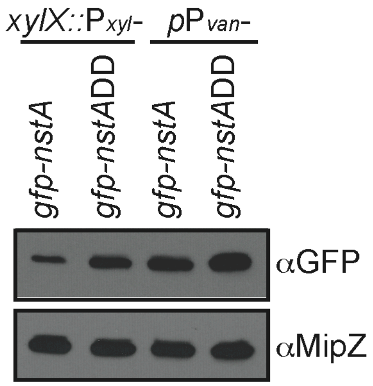

Figure S3. Immunoblots showing the steady state levels of GFP-NstA and GFP-NstADD expressed from the native *xylX* locus (*xylX*::P$_{xyl}$-*nstA* / *xylX*::P$_{xyl}$-*nstA*DD) or from the vanillate inducible promoter, P$_{van}$, on the plasmid *p*MT335 (*p*P$_{van}$-*nstA* / *p*P$_{van}$-*nstA*DD), probed with anti-GFP or anti-MipZ. The cells were treated with 0.3% xylose or 0.5 mM vanillate for 3 h. MipZ was used as the loading control.

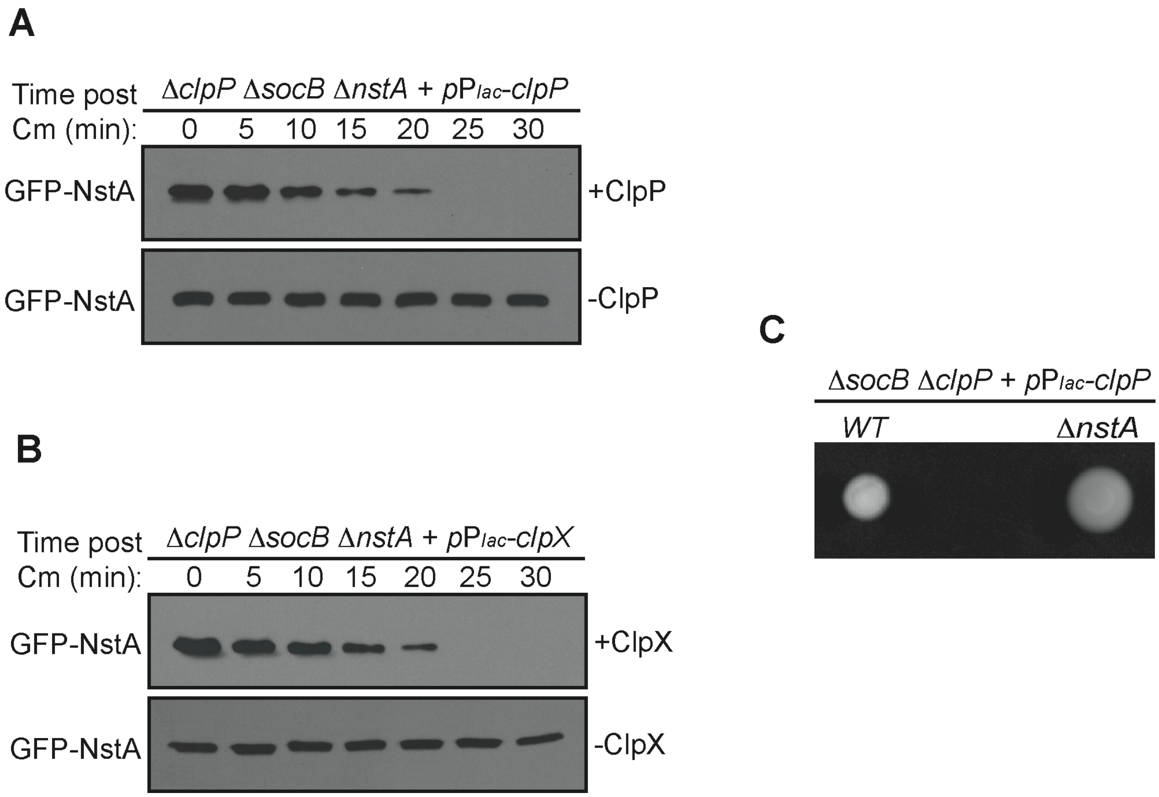

Figure S4. NstA regulation by the protease ClpXP. Immunoblots showing the difference in stability of GFP-NstA in the presence and absence of (A) ClpP, and (B) ClpX. The cells were grown in the presence or absence of IPTG to induce and deplete the expression of ClpP or ClpX. Expression of *gfp-nstA* was induced, with 0.3% xylose for 4 h, prior to the inhibition of translation by chrloramphenicol (Cm) treatment. The abundance of GFP-NstA was monitored over time as indicated. (C) Swarm assay plate showing the motility of cells in Figure 2D. The cells were not treated with IPTG.

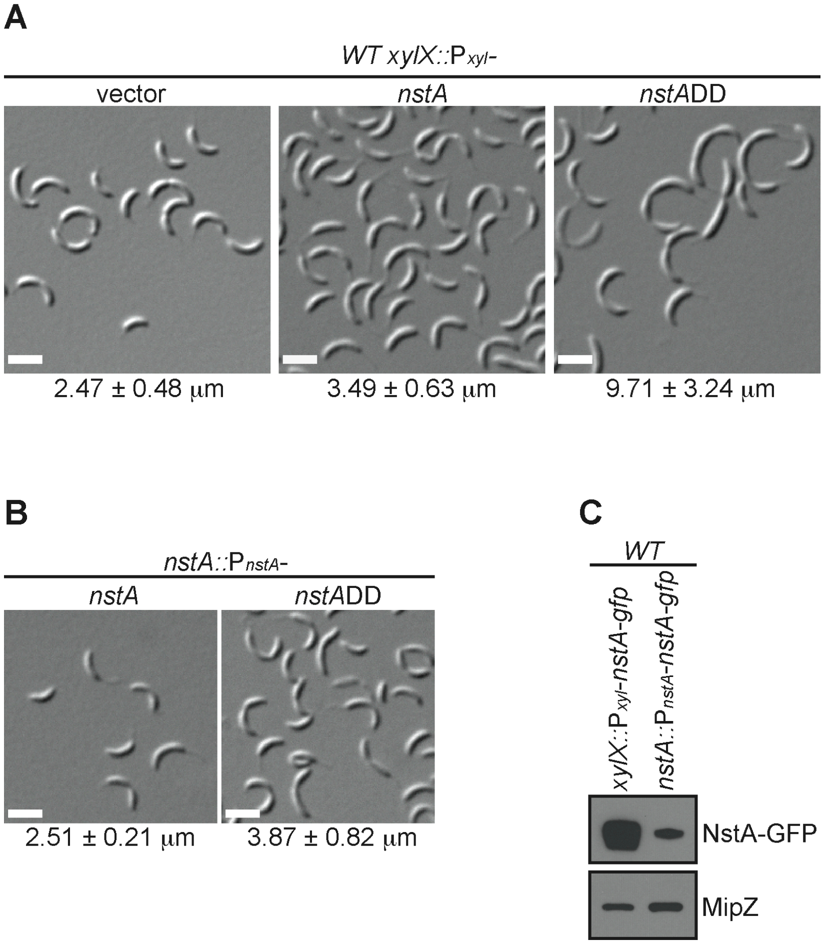

Figure S5. DIC microscopy images of *WT* cells harboring, (A) at the *xylX* locus, the vector (pXGFP4), *nstA* (*xylX*::P_{xyl}-*nstA*) or *nstA*DD (*xylX*::P_{xyl}-*nstA*DD), and (B) expressing *nstA* or *nstADD* from the native P_{nstA} locus. The cells in (A) were induced with 0.3% xylose for 6 h. Mean cell size, ±SD, of at least 200 cells is given at the bottom of the images. Scale bar, 2 μm. (C) Immunoblots depicting the levels of NstA-GFP expressed from the *xylX* locus (*xylX*::P_{xyl}-*nstA-gfp*) or the native *nstA* locus (*nstA*::P_{nstA}-*nstA-gfp*) on the chromosome. MipZ is used as the loading control.

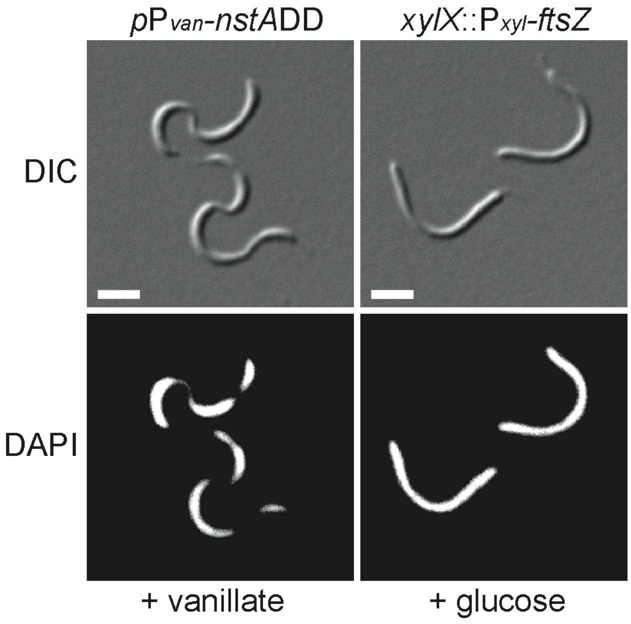

Figure S6. Chromosome free regions in cells overexpressing NstADD. DIC and fluorescence microscopic images of the DAPI stained *WT C. crescentus* overproducing *nstA*DD from the P_{van} promoter on *p*MT335, and *ftsZ* depleted cells, YB1585 (Wang et al. 2001). The cells were treated with 0.5 mM vanillate to induce the production of *nstA*DD or treated with 0.2% glucose to deplete the production of *ftsZ*, causing filamentation. Scale bar, 2 μm.

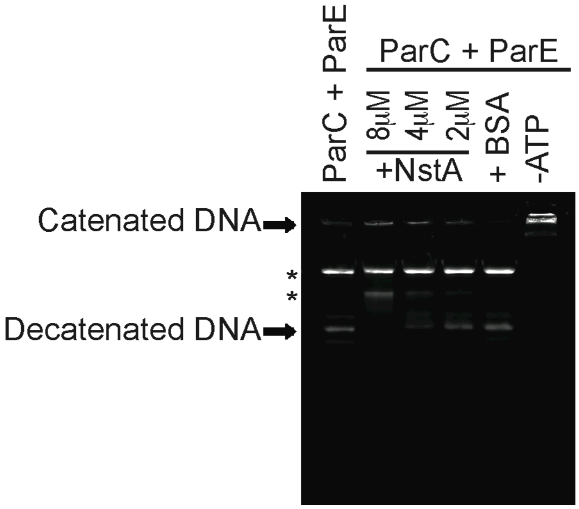

Figure S7. Inhibition of the decatenation activity of topo IV by NstA. Agarose gel, stained with ethidium bromide, depicting the decatenation assay described in Figure 3F. Asterisk (*) denotes incompletely decatenated products.

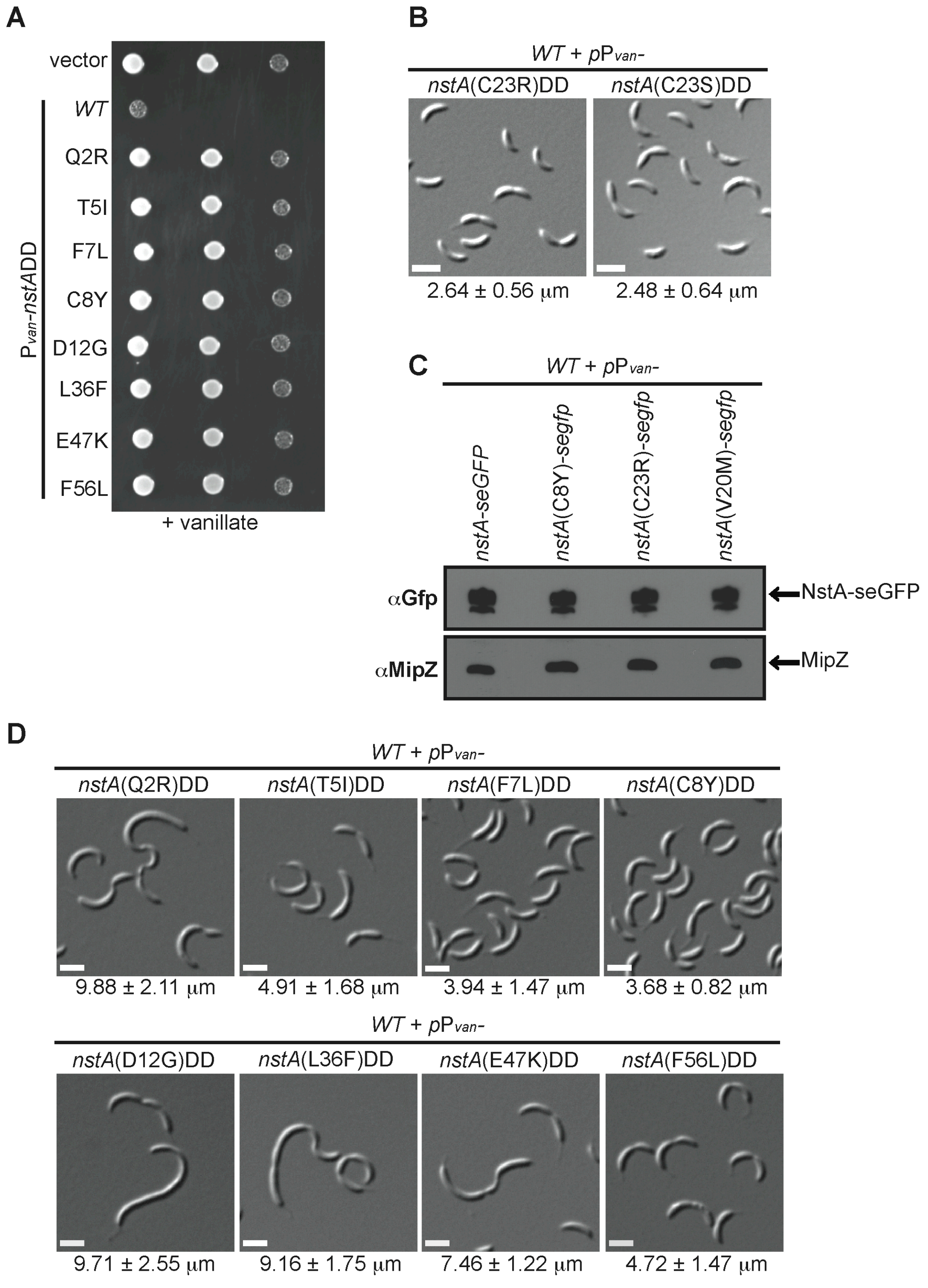

Figure S8. Activity suppressors of NstA. (A) Growth of *WT Caulobacter* overproducing *wild-type nstA*DD or the intragenic suppressor mutants of *nstA*DD, as indicated, from the P_{van} promoter on *p*MT335. Cells, as indicated, were diluted

five fold and spotted on media containing 0.5 mM vanillate. *WT* cells harboring the empty vector were used as the control. (B) DIC microscopy images of *WT* cells overexpressing *nstA*(C23R)DD or *nstA*(C23S)DD from the P_{van} promoter on *p*MT335. The cells were treated with 0.5 mM vanillate for 3 h. (C) Steady state immunoblot of *WT* cells overproducing NstA, NstA(C8Y), NstA(C23R) or NstA(V20M), with a C-terminal fusion of super folding GFP (seGFP), from the P_{van} promoter on *p*MT335, probed with anti-GFP or anti-MipZ. The cells were treated with 0.5 mM vanillate for 3 h. MipZ was used as the loading control. Note that NstA-seGFP is functional and behaves in similar manner as NstADD. (D) DIC microscopy images of cells in S8A. Mean cell size, ±SD, of at least 200 cells is given at the bottom of the images. Scale bar, 2 μm.

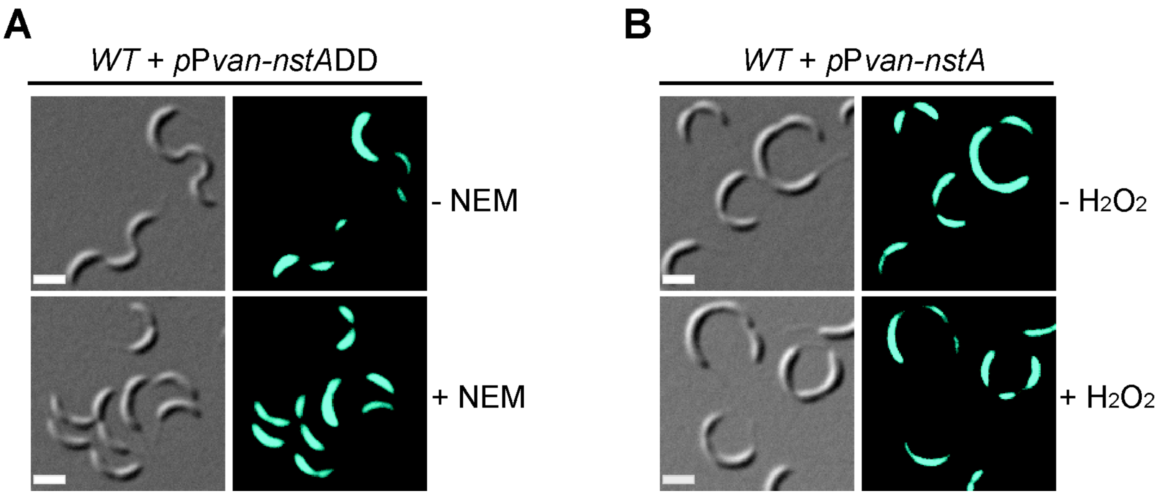

Figure S9. DIC and fluorescence microscopy images of *WT* cells overexpressing (A) *nstADD* and (B) *nstA* from the P_{van} promoter on the high copy vector, *p*MT335, and harboring the non-specific chromosome binding protein, HU, fused at the C-terminus with the cyan fluorescent protein (CFP) expressed from the native chromosomal locus (*hu*::*hu-cfp*)*.* The cells in (A) were treated with 0.25 mM vanillate in the presence or absence of 7.5 μM NEM for 2 h. The cells in (B) were treated with 0.5 mM vanillate for 6 h in the presence or absence of 40 μM H_2O_2. Scale bar, 2 μm.

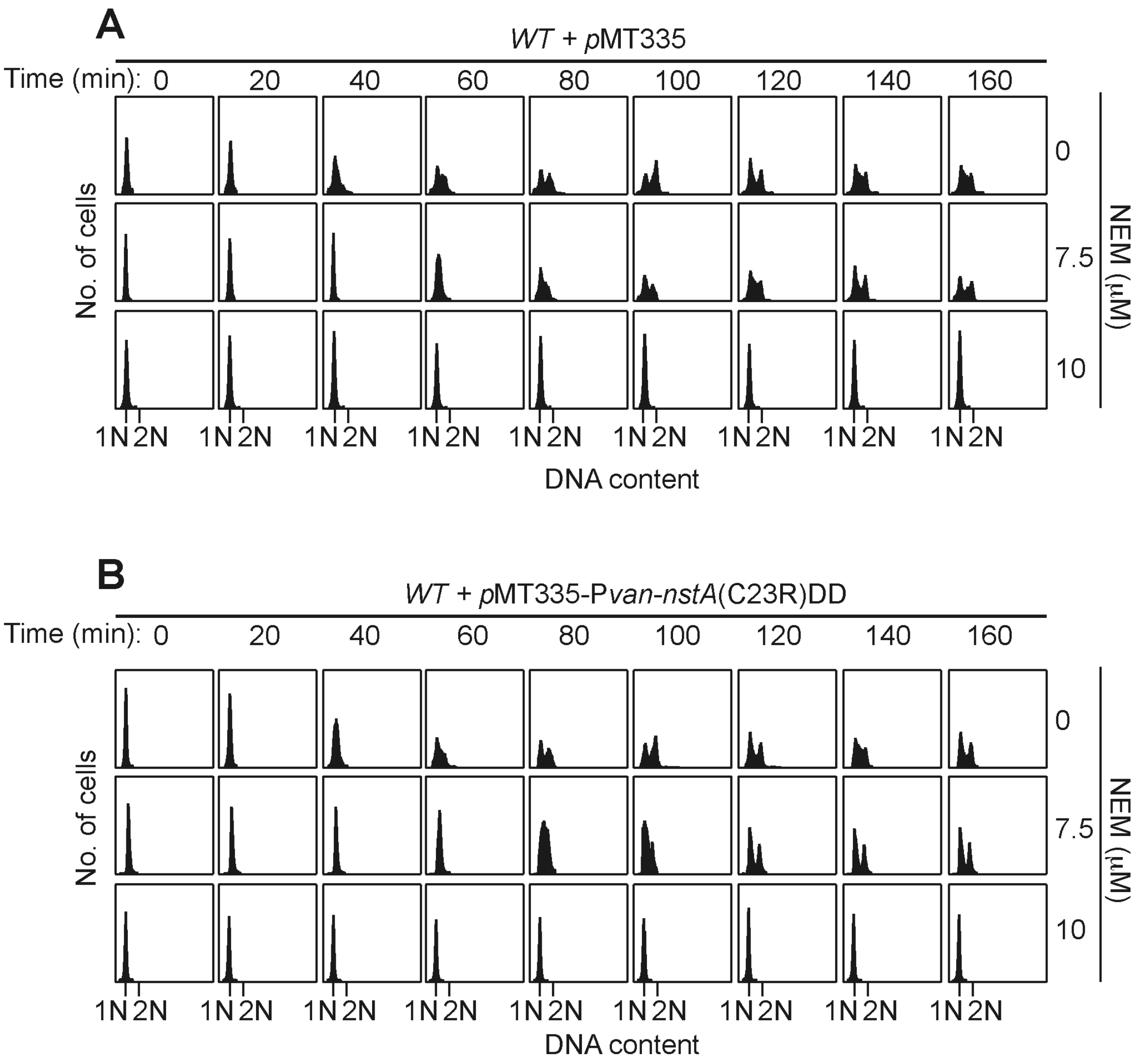

Figure S10. (A, B) Flow cytometry profiles showing the chromosome content, during the cell cycle, in synchronized population of *WT* cells harboring the plasmid *p*MT335 or cells expressing *nstA*(C23R)DD from the P_{van} promoter on *p*MT335, in the presence of 7.5 μM or 10 μM NEM as compared to the absence of NEM. The cells were pre-treated with 7.5 μM or 10 μM NEM prior to synchronization, and the synchronized population of cells, as indicated, were re-suspended in media containing vanillate with 7.5 μM or 10 μM NEM or without NEM.

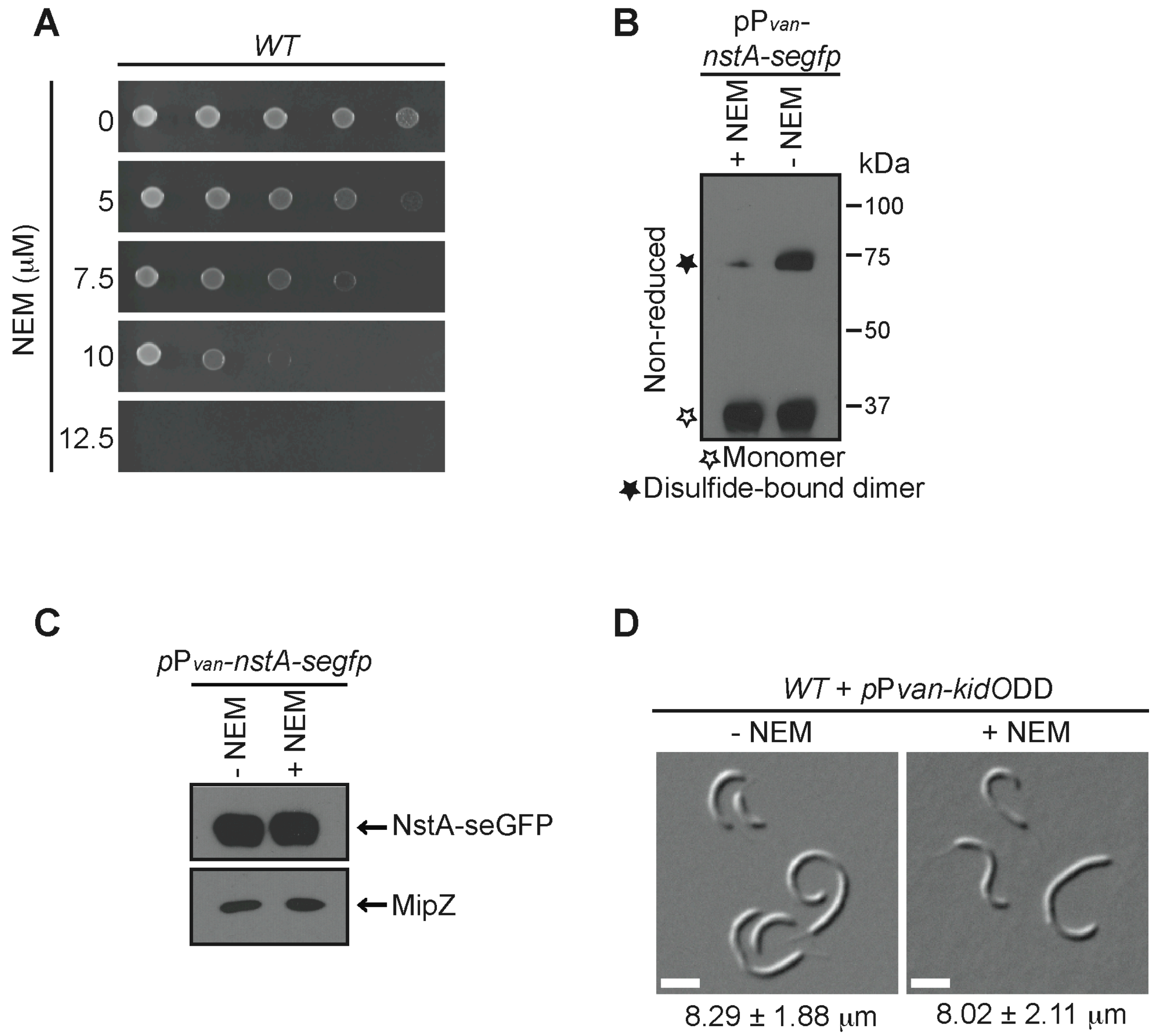

Figure S11. (A) Growth of *WT Caulobacter* in the presence of varying concentrations of NEM. Five fold dilution of *WT* cells were spotted on media containing different concentrations of NEM, as indicated. (B) Immunoblots of non-reducing SDS-PAGE of NstA-seGFP overproduced from the P_{van} promoter on *p*MT335 (*p*P_{van}-*nstA-segfp*) in the presence or absence of NEM. The cells were pre-treated with 7.5 μM NEM 2 h prior to the addition of 0.25 mM vanillate. (C) Immunoblots of cells overproducing *nstA-segfp* from the P_{van} promoter on *p*MT335, in the presence and absence of NEM. The cells were treated in the same manner as described in (B). Note that NstA-seGFP is functional and

behaves in similar manner as NstADD. (D) DIC microscopy images of *WT* cells overexpressing *kidO*DD, from P_{van} on *p*MT335, in the presence or absence of N-ethylmaleimide (NEM). The cells were treated in the same manner as described in (B).

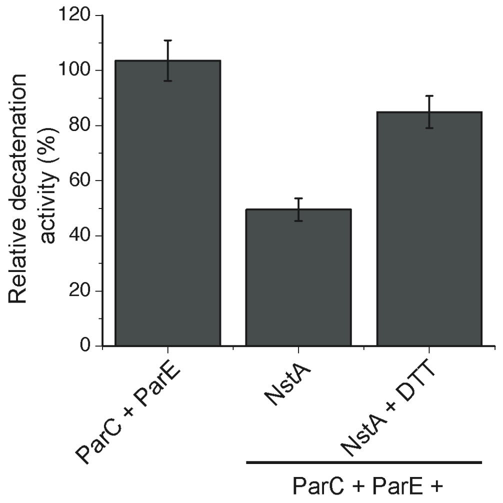

Figure S12. Dithiothreitol (DTT) inhibits the activity of NstA. Relative *in vitro* DNA decatenation activity of topo IV (ParC+ParE) in the presence of NstA and NstA+DTT. 6 mM DTT was used in the reaction. The data is average of 3 independent experiments ±SE.

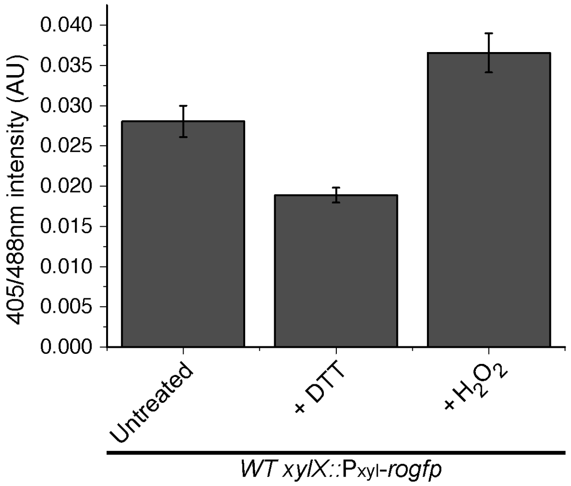

Figure S13. Analyses of the intracellular redox state. Data representing the 405/488 nm ratio of roGFP2 in *WT C. crescentus* treated with 20 mM dithiothreitol (DTT) or 2 mM hydrogen peroxide (H_2O_2). *rogfp2* was expressed from the chromosomal *xylX* locus (*xylX*::P_{xyl}-*rogfp2*). The cells were treated with 0.3% xylose to induce the production of roGFP2.

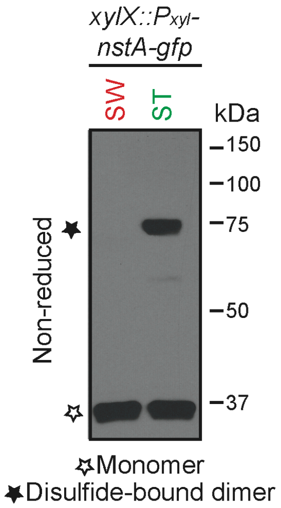

Figure S14. Effect of intracellular redox on the C23 dependent dimerization of NstA. Immunoblots of non-reducing SDS-PAGE of NstA-GFP in synchronized swarmer (SW) and stalked (ST) cell population in *WT Caulobacter* expressing *ntrD-gfp* from the chromosomal *xylX* locus (*xylX::*P$_{xyl}$-*nstA-gfp*). Note that the reduced cytoplasm of the SW cells does not allow the cysteine dependent dimerization of NstA-GFP. The dimerized NstA-GFP in the ST cell population is from the early ST cells whose cytoplasm is oxidized during cell cycle.

SUPPLEMENTAL TABLE

Table S1. Targets whose promoters are precipitated by GcrA and m6A antibodies, and harbor upstream 5'-GANTC-3' sites. Derived from the ChIP-Seq data of Fioravanti et al. 2013.

Gene number	Annotation
CCNA_00007	SSU ribosomal protein S20P
CCNA_00020	Hypothetical Protein
CCNA_00055	Ferric Uptake Protein
CCNA_00267	MutT like Protein
CCNA_00291	PhoR
CCNA_00321	LSU Ribosomal Protein, L21P
CCNA_00390	ADP-Heptose-LPS Heptosyl Transferase
CCNA_00464	Undecaprenyl phosphate sugar phosphotransferase related protein
CCNA_00504	23S rRNA Methyl Transferase
CCNA_00550	LacI Family Transcriptional Regulator
CCNA_00551	Hypothetical Protein
CCNA_00665	GAF Family Sensor Histidine Kinase
CCNA_00697	
CCNA_00793	Phosphatidylglycerol Glycosyl Transferase
CCNA_00922	ClpB
CCNA_00977	Transposase, IS4/5 Family
CCNA_01108	Nucleoside Diphosphate Sugar Epimerase
CCNA_01163	Ice Nucleation Protein
CCNA_01181	Conserved Hypothetical Protein
CCNA_01221	Acyl Carrier Protein
CCNA_01297	23S rRNA GM2251 Methyl Transferase
CCNA_01417	5-Aminolevulinic Acid Synthase
CCNA_01467	Cytochrome cbb3 oxidase subunit ccoN
CCNA_01523	Acetyl Transferase FlmH
CCNA_01524	FlbA
CCNA_01532	FlaY
CCNA_01542	Ice Nucleation Protein
CCNA_01556	LacI Family Transcriptional Regulator
CCNA_01637	Topoisomerase IV subunit A
CCNA_01684	PhenylAlanine-4-Hydroxylase
CCNA_01686	Exopolyphosphatase GppA
CCNA_01766	CsgG related Curli Protein
CCNA_01853	Hypothetical Protein

CCNA_01901	Lipoprotein
CCNA_02003	DnaE
CCNA_02033	NADH-Quinone Oxidoreductase A chain
CCNA_02041	ClpP
CCNA_02096	CusF Family Protein
CCNA_02126	Conserved Hypothetical Protein
CCNA_02200	Cytochrome c Family Protein
CCNA_02246	MipZ
CCNA_02287	TetR Family Transcriptional Regulator
CCNA_02401	SalR Family Ligand Binding Transcriptional Regulator
CCNA_02416	HU, DNA Binding Protein
CCNA_02428	Translation Initiation Factor, IF-1
CCNA_02436	Hypothetical Protein
CCNA_02449	Conserved Hypothetical Protein
CCNA_02528	PlsY like Acyl Phosphate Glycerol-3-Phosphate Acyl Transferase
CCNA_02576	Phosphoribosylamidoimidazole-Succinocarboxamide Synthase
CCNA_02623	FtsZ
CCNA_02726	Gamma Carbonic Anhydrase Family Protein
CCNA_02779	LysM Family Peptidoglycan Binding Protein
CCNA_02782	Hypothetical Protein
CCNA_02805	Hypothetical Protein
CCNA_03062	Cell wall Hydrolase Family Protein
**CCNA_03091**	**NstA**
CCNA_03092	Cytochrome P 450 Family Protein
CCNA_03130	CtrA
CCNA_03371	Bacterioferritin
CCNA_03406	RpsU-SSU Ribosome Protein S21P
CCNA_03516	ProtohemeIX Farnesyl Transferase, CoxE
CCNA_03518	Cytochrome c Oxidase Polypeptide II, CoxB
CCNA_03598	DivL
CCNA_03609	Outer Membrane Protein
CCNA_03689	Alanine Dehydrogenase
CCNA_03736	YGGT Family Membrane Protein
CCNA_03813	CreS
CCNA_03830	Multidrug Resistance Protein A Efflux Pump

Table S2. Cell length, at various time intervals, of cells overexpressing *nstA* or *nstA*DD from P_{van} on *p*MT335.

Strain	Cell length post induction with vanillate (µm)			
	**3 hr**	**6 hr**	**9 hr**	**12 hr**
WT + *p*P_{van}-*nstA*	3.21 ± 0.75	5.93 ± 1.22	7.74 ± 1.85	10.19 ± 1.85
WT + *p*P_{van}-*nstA*DD	14.84 ± 3.25	18.75 ± 3.72	22.65 ± 4.23	Lysed

SUPPLEMENTAL REFERENCE

Aakre CD, Phung TN, Huang D, Laub MT. 2013. A bacterial toxin inhibits DNA replication elongation through a direct interaction with the beta sliding clamp. *Mol Cell* **52**: 617-628.

Bendezu FO, Hale CA, Bernhardt TG, de Boer PA. 2009. RodZ (YfgA) is required for proper assembly of the MreB actin cytoskeleton and cell shape in E. coli. *EMBO J* **28**: 193-204.

Domian IJ, Quon KC, Shapiro L. 1997. Cell type-specific phosphorylation and proteolysis of a transcriptional regulator controls the G1-to-S transition in a bacterial cell cycle. *Cell* **90**: 415-424.

Edelheit O, Hanukoglu A, Hanukoglu I. 2009. Simple and efficient site-directed mutagenesis using two single-primer reactions in parallel to generate mutants for protein structure-function studies. *BMC biotechnology* **9**: 61.

Figge RM, Divakaruni AV, Gober JW. 2004. MreB, the cell shape-determining bacterial actin homologue, co-ordinates cell wall morphogenesis in Caulobacter crescentus. *Mol Microbiol* **51**: 1321-1332.

Fioravanti A, Fumeaux C, Mohapatra SS, Bompard C, Brilli M, Frandi A, Castric V, Villeret V, Viollier PH, Biondi EG. 2013. DNA binding of the cell cycle transcriptional regulator GcrA depends on N6-adenosine methylation in Caulobacter crescentus and other Alphaproteobacteria. *PLoS Genet* **9**: e1003541.

Gonzalez D, Collier J. 2013. DNA methylation by CcrM activates the transcription of two genes required for the division of Caulobacter crescentus. *Mol Microbiol* **88**: 203-218.

Gutscher M, Pauleau AL, Marty L, Brach T, Wabnitz GH, Samstag Y, Meyer AJ, Dick TP. 2008. Real-time imaging of the intracellular glutathione redox potential. *Nat Methods* **5**: 553-559.

Holtzendorff J, Hung D, Brende P, Reisenauer A, Viollier PH, McAdams HH, Shapiro L. 2004. Oscillating global regulators control the genetic circuit driving a bacterial cell cycle. *Science* **304**: 983-987.

Pedelacq JD, Cabantous S, Tran T, Terwilliger TC, Waldo GS. 2006. Engineering and characterization of a superfolder green fluorescent protein. *Nat Biotechnol* **24**: 79-88.

Radhakrishnan SK, Pritchard S, Viollier PH. 2010. Coupling Prokaryotic Cell Fate and Division Control with a Bifunctional and Oscillating Oxidoreductase Homolog. **18**: 90-101.

Radhakrishnan SK, Thanbichler M, Viollier PH. 2008. The dynamic interplay between a cell fate determinant and a lysozyme homolog drives the asymmetric division cycle of Caulobacter crescentus. *Genes Dev* **22**: 212-225.

Schmalen I, Reischl S, Wallach T, Klemz R, Grudziecki A, Prabu JR, Benda C, Kramer A, Wolf E. 2014. Interaction of circadian clock proteins CRY1 and PER2 is modulated by zinc binding and disulfide bond formation. *Cell* **157**: 1203-1215.

Thanbichler M, Iniesta AA, Shapiro L. 2007. A comprehensive set of plasmids for vanillate- and xylose-inducible gene expression in Caulobacter crescentus. *Nucleic Acids Res* **35**: 24.

Thanbichler M, Shapiro L. 2006. MipZ, a spatial regulator coordinating chromosome segregation with cell division in Caulobacter. *Cell* **126**: 147-162.

Wang SC, Shapiro L. 2004. The topoisomerase IV ParC subunit colocalizes with the Caulobacter replisome and is required for polar localization of replication origins. *Proc Natl Acad Sci U S A* **101**: 9251-9256.

Wang Y, Jones BD, Brun YV. 2001. A set of ftsZ mutants blocked at different stages of cell division in Caulobacter. *Mol Microbiol* **40**: 347-360.

Ward D, Newton A. 1997. Requirement of topoisomerase IV parC and parE genes for cell cycle progression and developmental regulation in Caulobacter crescentus. *Mol Microbiol* **26**: 897-910.
